# Magnetic resonance searches for Earth-sourced exotic nuclear spin–dependent interactions

**DOI:** 10.1126/sciadv.aeg0974

**Published:** 2026-09-16

**Authors:** Yuanhong Wang, Xingming Huang, Jiaxi Li, Wenzhong Wang, Xiang Kang, Yuanxing Liu, Lei Cong, Dmitry Budker, Min Jiang, Xinhua Peng

**Affiliations:** ^1^ Laboratory of Spin Magnetic Resonance, School of Physical Sciences, Anhui Province Key Laboratory of Scientific Instrument Development and Application, University of Science and Technology of China, Hefei 230026, China.; ^2^ Laboratory of Seismology and Physics of Earth’s Interior, School of Earth and Space Sciences, University of Science and Technology of China, Hefei, Anhui 230026, China.; ^3^ National Geophysical Observatory at Mengcheng, University of Science and Technology of China, Hefei 230026, China.; ^4^ Chinese Academy of Sciences Center for Excellence in Comparative Planetology, USTC, Hefei, Anhui 230026, China.; ^5^ Institute of Quantum Sensing and School of Physics, Zhejiang University, Hangzhou 310027, China.; ^6^ Excellence Cluster PRISMA++, Johannes Gutenberg-Universität Mainz, 55128 Mainz, Germany.; ^7^ Helmholtz-Institut Mainz, 55099 Mainz, Germany.; ^8^ GSI Helmholtzzentrum für Schwerionenforschung GmbH, 64291 Darmstadt, Germany.; ^9^ Department of Physics, University of California, Berkeley, Berkeley, CA 94720, USA.; ^10^ Hefei National Laboratory, University of Science and Technology of China, Hefei 230088, China.

## Abstract

Searches for physics beyond the standard model motivate investigations into ultralight exotic bosons, which may mediate long-range spin-spin interactions. Here, we develop an unreported method based on nuclear magnetic resonance (NMR) performed at the surface of Earth to search for exotic interactions sourced by astrophysical bodies. The key aspects of the work are modeling the internal nuclear spin polarization of Earth and developing an NMR detector to sense the exotic fields produced by such polarization. We create a distribution map of underground polarized nucleons by combining nuclear physics and geophysics data, identifying approximately $5.3\times 10^{37}$ polarized geoprotons. The searches are enabled by a dual-xenon-isotope NMR sensor combined with phase cycling techniques, which suppresses systematic errors by more than 200-fold compared with a measurement without using these reversals. As a result, the experiment can detect exotic fields corresponding to equivalent static magnetic fields at the femtotesla level. Our work sets the most stringent limits on axion-mediated couplings $g_{P}^{n}g_{P}^{p}$, as well as on *Z*′ boson–mediated couplings $g_{A}^{n}g_{A}^{p}$ and $g_{A}^{n}g_{V}^{p}$. In particular, the limits on $g_{A}^{n}g_{A}^{p}$ improve upon previous bounds by more than 15 orders of magnitude for the force range beyond 4.0 kilometers. Our technique opens an avenue for a wide range of terrestrial and space-based quantum sensors in studying macroscopic-scale fundamental physics.

## INTRODUCTION

Numerous theoretical frameworks predict the existence of new bosons beyond the standard model of particle physics (*1*–*3*), such as axions, axion-like particles, and *Z*′ bosons. For example, axions can generically arise in theories at ultrahigh energies (*4*, *5*), including string theory, grand unified theories, and models with extra dimensions. They represent promising candidates to resolve several outstanding puzzles in physics (*1*, *6*), including the dark matter problem and the strong CP problem in quantum chromodynamics. The exchange of such bosons can induce new interactions between standard model fermions (*7*–*9*), distinct from the four known fundamental forces; see the recent review (*10*). First proposed by Moody and Wilczek (*7*) and later extended by Dobrescu and Mocioiu (*8*), these exotic interactions are typically spin dependent and can exhibit interaction ranges spanning from subatomic (*10*–*14*) to astrophysical scales (*15*–*20*). Crucially, studying these interactions allows probes of a wide range of exotic boson masses without requiring exhaustive mass scans or assuming that these bosons constitute dark matter (*21*–*23*).

Experimental searches for exotic feeble interactions typically use paired particle ensembles, with one serving as a spin or mass source that generates an exotic field and the other acting as a detector to measure it. To improve the sensitivity of these searches, recent efforts (*24*–*27*) have primarily developed novel detectors or enhanced existing detector sensitivities. We explore a complementary direction by first introducing a new class of spin sources. In particular, we propose and use Earth itself as a polarized, planetary-scale nucleon spin source, thereby substantially enhancing the available signal strength, as detailed later. Existing searches using nucleon (neutron or proton) spin sources typically rely on controlled laboratory systems such as free neutrons, noble-gas nuclei, or alkali-metal atoms (*28*–*31*). While astrophysical bodies like the Sun, Earth, and Moon have recently been used as electron-spin or mass sources (*15*–*20*), their vast internal resources of polarized nucleons remain unexplored because of substantial challenges. For example, using Earth as a natural nucleon spin source requires a comprehensive understanding of its internal structure and a precise model of the spatially distributed polarized nucleons. This task is inherently challenging, as it necessitates interdisciplinary inputs from nuclear physics and geophysics.

The detection of exotic signals from planetary-scale objects presents further challenges because of their typically low frequencies or lack of modulation (*15*, *19*). For example, exotic fields originating from Sun or Moon show daily modulation for detectors on Earth (*20*), while signals from Earth itself remain constant (*15*–*18*). This stands in stark contrast to laboratory-scale searches, where source modulation (e.g., from rotating SmCo_5_ or Bi_4_Ge_3_O_12_ sources) can be precisely controlled at frequencies of tens to hundreds of hertz (*24*, *32*–*35*). The low-frequency or static nature of planetary signals imposes severe demands on sensing techniques, as suppressing low-frequency noise and constant systematic offsets is considerably more difficult than rejecting high-frequency interference (*36*). At present, many sensors exhibit low sensitivity to low-frequency or static signals. For example, state-of-the-art detectors based on spin exchange relaxation–free magnetometers (*33*, *34*, *37*) or spin-based amplifiers (*30*, *31*), while achieving sensitivities near 10 fT/Hz^1/2^ around 10 Hz, see their performance degrade to worse than 1 pT/Hz^1/2^ at frequencies below 1 Hz. Dual noble-gas comagnetometers (*19*) and Hg-based comagnetometers (*38*) for exotic-interaction searches also exhibit sensitivities worse than 500 fT/Hz^1/2^ for static signal measurements. Furthermore, self-compensated comagnetometers (*35*, *39*) designed for low-frequency operation are effective for modulated signals and cannot perform absolute measurements of static fields. Consequently, search methods suitable for constant planetary-scale exotic signals remain underdeveloped.

In this work, we introduce a surface nuclear magnetic resonance (NMR) technique designed to probe exotic spin-spin couplings originating from the spin-polarized nucleons within Earth. Initially, we create a distribution map of polarized protons and neutrons inside Earth, based on the mantle mineral composition and the abundance of elements with nonzero nuclear spin. Our analysis shows that Earth contains more than $2.2\times 10^{48}$ valence protons and $2.8\times 10^{48}$ valence neutrons, with more than 1 in every 100 billion nucleons polarized parallel to the geomagnetic field. This results in approximately $5.3\times 10^{37}$ polarized protons and $2.2\times 10^{37}$ polarized neutrons, surpassing existing laboratory nucleon sources by more than $10^{16}$ times. Yet, detecting static exotic-interaction signals from these “geonucleons” remains a challenge. To overcome this, we develop a surface-NMR detector based on X129e−X131e dual noble gases, exploiting their different (and opposite-sign) gyromagnetic ratios to reduce low-frequency common-mode magnetic noise by nearly 100-fold. In addition, by implementing a phase-cycling procedure where the bias field direction and detector orientation are systematically altered, we effectively isolate the exotic signals, thus reducing systematic error by more than 200-fold compared to a nonreversing measurement. After subtracting the Earth-rotation effect, we measure a static exotic field equivalent to −4.1±12.8 fT. By combining our geonucleon model with this surface-NMR method, we explore a previously unexplored parameter space of axion-mediated coupling $g_{P}^{n}g_{P}^{p}$. Furthermore, we establish the most stringent limits on $g_{A}^{n}g_{A}^{p}$ and $g_{A}^{n}g_{V}^{p}$ for *Z*′ boson–mediated interactions over force ranges exceeding 10^4^ m, improving previous constraints by up to 15 orders of magnitude. Our Earth nucleon model adds an unreported spin source to the exotic-interaction research “toolbox” and will be made openly available for future research. Moreover, our approach can be extended to other astrophysical bodies like Jupiter, which is expected to contain even more polarized nucleons, potentially enhancing sensitivity by an additional six orders of magnitude.

## RESULTS

### Dedicated NMR searches using Earth-sourced nucleon

**Fig. 1.**
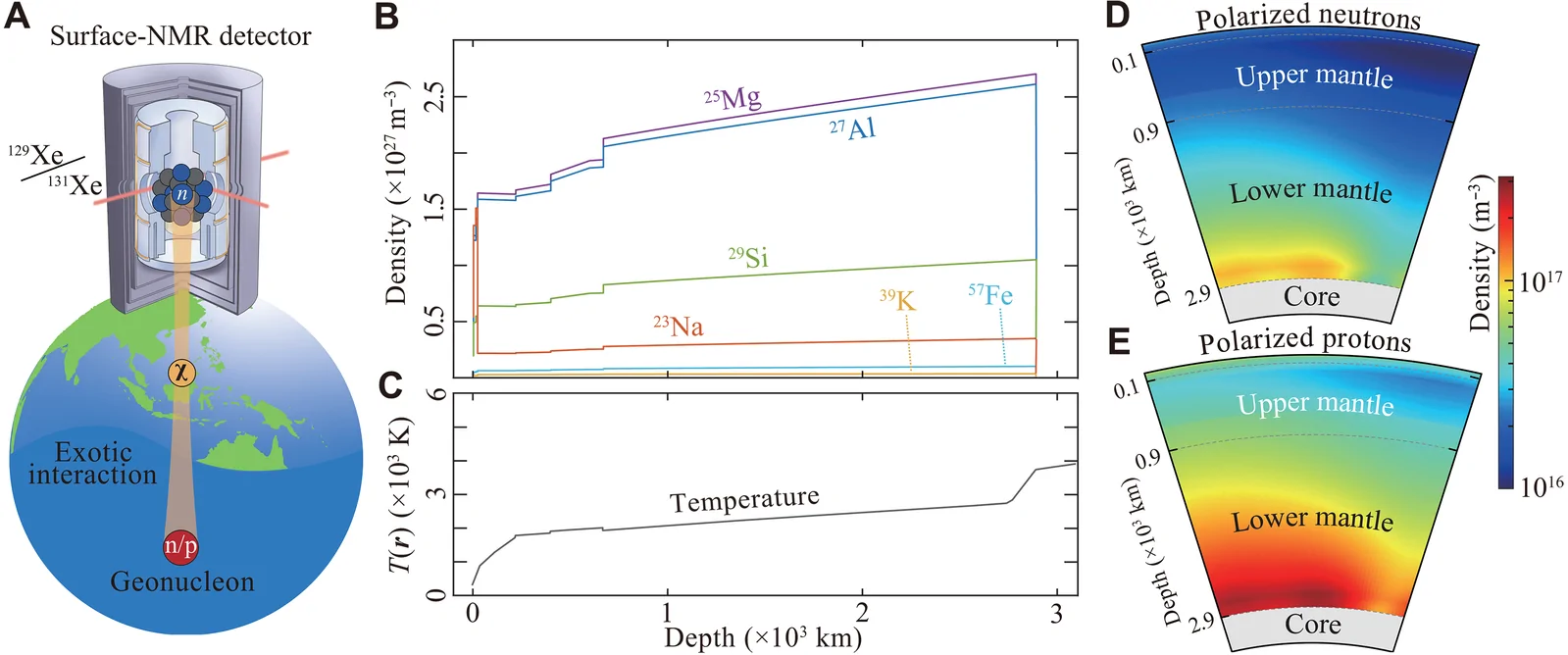
Searches for exotic SSIs between Earth’s polarized nucleons and a surface-NMR detector. (**A**) Visualization of surface-NMR approach to search for exotic SSIs. At the core of our surface-NMR detector are spatially overlapping ensembles of ^129^Xe and ^131^Xe atoms. The nuclear spins of these isotopes are both primarily carried by their valence neutrons. The newly developed surface-NMR approach targets SSIs mediated by an exotic boson χ between subterranean polarized nucleons (geoneutrons and geoprotons, n/p) and the polarized valence neutrons (n) of the detector, where χ can be axions or *Z*′ bosons. (**B**) Average isotope density as a function of Earth’s depth. Isotope densities are calculated from corresponding element densities and natural abundances. (**C**) Temperature profile as a function of Earth’s depth. (**D** and **E**) Polarized neutron- and proton-spin density, respectively, in a 60° sector of the meridian plane at 117.17°E beneath the detector position (Hefei: 117.17°E, 31.83°N).

We develop a surface-NMR technique to investigate exotic spin-spin interactions (SSIs). Unlike traditional NMR experiments, which place samples inside a detector to study scalar spin-spin couplings between nuclei within molecules (*14*, *40*–*44*), our approach positions the detector at the surface of the macroscopic sample (i.e., Earth) under investigation. It probes the exotic interactions between the subsurface nucleon ensembles and the detector, mediated by new bosons—termed surface NMR, as illustrated in Fig. 1A. Thereby, we expand the SSI-search experimental scope from laboratory-scale spin sources, such as microscopic samples, spin-polarized gases, or SmCo_5_, to entire Earth. In this work, we demonstrate that Earth is abundant in polarized protons and neutrons, making it an ideal nuclear spin source to substantially enhance the exotic SSI signals.

We emphasize that using Earth as a nuclear spin source presents two considerable challenges: accurately mapping the distribution of underground spin-polarized nucleon density and detecting the resulting nonmodulated exotic SSI signals. About a decade ago, Hunter *et al*. (*15*) constructed a geoelectron model of Earth, followed by a series of related studies (*17*, *18*, *45*). However, nuclear spins have been systematically neglected, largely because of the associated computational complexities. To address this gap, we develop a comprehensive model of Earth’s internal polarized nucleon spins, constructing detailed distribution maps of polarized geoprotons $\rho_{p}\left(r\right)$ and geoneutrons $\rho_{n}\left(r\right)$ at every position r throughout Earth. This modeling process involves tackling three primary complexities: first, identifying isotopes *i* within Earth that have net neutron or proton spins and determining the fractional contribution $\sigma_{n}^{i}$ or $\sigma_{p}^{i}$ of nucleon spins to their total nuclear spin $I_{i}$; second, obtaining the global isotopic distribution density $\rho_{i}\left(r\right)$ at every location r; and third, calculating the magnitude and orientation of the thermal spin polarization $P_{i}\left(r\right)$ of isotopic nuclei induced by the geomagnetic field. These input parameters require an integration of nuclear physics and geophysics, as discussed in Materials and Methods.

We focus on three types of exotic SSIs between geonucleons and neutrons in our surface-NMR detector. The exchange of a new boson χ between two spin-1/2 fermions can result in 16 independent new interactions (*8*). Among these, three SSIs denoted as $V_{3}$, $V_{2}$, and $V_{11}$ are of particular interest because of their high sensitivity in probing specific couplings: pseudoscalar/pseudoscalar ($g_{P}g_{P}$) with χ=axion, and axial-vector/axial-vector ($g_{A}g_{A}$) and axial-vector/vector ($g_{A}g_{V}$) with $\chi =Z^{\prime }\;\text{boson}$. These interactions are characterized by potentials (*10*, *46*, *47*) (where ℏ=c=1)

$$\begin{array}{c} {V_{3}|}_{\text{axion}}=-g_{P}^{n}g_{P}^{p}\left[\left({\hat{\sigma }}_{n}\cdot {\hat{\sigma }}_{p}\right),\left(\frac{1}{\lambda r^{2}}+\frac{1}{r^{3}}\right)\right.\\ -\left.\left({\hat{\sigma }}_{n}\cdot \hat{r}\right),\left({\hat{\sigma }}_{p}\cdot \hat{r}\right),\left(\frac{1}{\lambda^{2}r}+\frac{3}{\lambda r^{2}}+\frac{3}{r^{3}}\right)\right]\frac{e^{-r/\lambda }}{16\pi m_{n}^{2}} \end{array}$$ (1)

$$\begin{array}{c} {V_{2}+V_{3}|}_{Z^{\prime }}=-g_{A}^{n}g_{A}^{p}\left[\left({\hat{\sigma }}_{n}\cdot {\hat{\sigma }}_{p}\right),\left(\frac{1}{r}+\frac{\lambda }{r^{2}}+\frac{\lambda^{2}}{r^{3}}\right)\right.\\ -\left.\left({\hat{\sigma }}_{n}\cdot \hat{r}\right),\left({\hat{\sigma }}_{p}\cdot \hat{r}\right),\left(\frac{1}{r}+\frac{3\lambda }{r^{2}}+\frac{3\lambda^{2}}{r^{3}}\right)\right]\frac{e^{-r/\lambda }}{4\pi } \end{array}$$ (2)

$${V_{11}|}_{Z^{\prime }}=-g_{A}^{n}g_{V}^{p}\left[\left({\hat{\sigma }}_{n}\times {\hat{\sigma }}_{p}\right)\cdot \hat{r}\right]\left(\frac{1}{\lambda r}+\frac{1}{r^{2}}\right)\frac{e^{-r/\lambda }}{8\pi m_{n}}$$ (3)

where ${\hat{\sigma }}_{n}$ and ${\hat{\sigma }}_{p}$ are the spin vectors of the surface-detector neutrons and geoprotons, respectively; $m_{n}$ denotes the nucleon mass; $\hat{r}$ is the unit vector pointing from neutrons to geoprotons; *r* is their separation; and $\lambda =1/m_{\chi }$ is the force range, with $m_{\chi }$ being the mass of the mediator boson χ.

To address the challenge of detecting nonmodulated exotic signals originating from Earth, we develop a surface detector based on a ^129^Xe–^131^Xe NMR system, combined with a developed NMR phase cycling technique to maximize the suppression of constant systematic errors. In this setup, the xenon nuclear spins within the detector would sense energy shifts induced by the polarized geonucleons via the exotic SSI potentials described in Eqs. 1 to 3, analogous to the Zeeman interaction. These energy shifts manifest as pseudomagnetic fields resulting from integrating SSI contributions throughout Earth’s interior. For example, the interaction $V_{2}$ between geoprotons and detector neutrons produces a pseudomagnetic field given by $B_{2}^{\text{SSI}}=-g_{A}^{n}g_{A}^{p}\int \rho_{p}\left(r\right)\cdot e^{-r/\lambda }{\left(4{\pi \mu }_{N}r\right)}^{-1}\;dr$, with its magnitude scaled appropriately for ^129^Xe or ^131^Xe sensing atoms. Our analysis in Materials and Methods shows that Earth-induced exotic interactions behave as static pseudomagnetic fields. Taking advantage of common-mode signal differential in the ^129^Xe–^131^Xe system and noncommon-mode noise cancellation via the phase cycling technique, we suppress systematic errors below the level of statistical error, enabling clear SSI signal extraction. Details on the elimination of nonmodulation systematic errors are provided in Materials and Methods.

### Polarized geonucleon density map

We create a distribution map of polarized geonucleon density $\rho_{n/p}\left(r\right)$ through an interdisciplinary integration of results from nuclear physics and geophysics. According to the nuclear shell model, nuclear spin arises primarily from unpaired odd nuclei (valence protons or neutrons), as paired nucleons exhibit mutually canceled spins (*48*). In Earth, proton spins are mainly contributed by odd-proton nuclei such as the abundant isotope ^27^Al, along with minor quantities of ^23^Na and ^39^K present in the crust. Conversely, isotopes with odd neutrons are relatively scarce; for example, ^17^O constitutes only 0.04% of all oxygen. On the basis of Earth’s composition and isotopic relative abundances suggested by the geoscience community (*49*–*51*), we identify ^25^Mg, ^29^Si, and ^57^Fe as the dominant contributors to neutron spins, listed in order of significance. Using the nuclear Schmidt model, we further calculate the valence nucleon spin projection $\sigma_{n/p}^{i}=<S_{n/p}\cdot I_{i}>/I_{i}/\left(I_{i}+1\right)$ in Materials and Methods for each isotope *i* with nuclear spin $I_{i}$. On the basis of the nucleon spin projection, we introduce the effective net nucleon number per isotope atom, $2I_{i}\sigma_{n/p}^{i}$, listed in column 12 of Table 1. This parameter accounts for the fact that different isotopes may contribute with opposite signs depending on the orientation of the valence nucleon spin relative to the total nuclear spin. For example, a ^27^Al atom with its valence proton in the $3d_{5/2}$ ground state effectively contributes +1 net proton; whereas a ^23^Na atom with its valence proton in the $3d_{3/2}$ state yields an effective net proton of −0.6, indicating an opposing proton spin orientation relative to the nuclear spin. When summing over all isotopes, these signed contributions naturally incorporate the cancellation or enhancement effects in the total polarized nucleon density. We emphasize that some major elements, such as oxygen and calcium, are not listed in Table 1 because of their extremely low abundances of nonzero-spin isotopes, rendering their contributions negligible (see Materials and Methods for details).

**Table 1.** Characteristics of key isotopes (*i*) that provide polarized spins within Earth. The spin type specifies the primary spin carrier, electron (e), neutron (n), or proton (p). The subsequent columns under key mantle minerals and crustal rocks list the percentage abundance of the isotope within each specific mineral phase—namely, K-feldspar (Kfs), plagioclase (Pl), biotite (Bt), garnet (Grt), olivine (Ol), bridgmanite (Bdg), perovskite (Prv), and ferropericlase (Fp)—with 0% indicating its absence or negligible presence. The total numbers (num.) of atoms provide the calculated global quantity of each species $\int \rho_{i}\left(r\right)\;dr$. For a nucleus with spin type n or p, the parameter $2I_{i}\sigma_{n/p}^{i}$ gives the effective net nucleon count per atom for isotope *i*, serving as the key factor in computing the polarized spin number following Eq. 4.

		Key mantle minerals and crustal rocks.								Total Num. of atoms	$2I\sigma_{n/p}$	Polarized spin num.	Work
Isotope	Spin Type	Kfs	Pl	Bt	Grt	Ol	Bdg	Prv	Fp				
Fe^2+^/Fe^3+^	e	0%	0%	0.21%	7.60%	6.98%	53.36%	1.05%	13.85%	$∼10^{49}$	–	$6.4\times 10^{41}$	(*15*)
^57^Fe	n	0%	0%	0.23%	8.15%	7.49%	53.93%	1.12%	14.86%	$7.4\times 10^{46}$	−1/3	$-3.9\times 10^{34}$	Thiswork
^25^Mg	n	0%	0%	0.03%	1.54%	17.79%	46.06%	0.96%	18.06%	$2.0\times 10^{48}$	+1	$+1.4\times 10^{37}$	
^29^Si	n	0.19%	0.94%	0.05%	3.24%	7.26%	68.06%	11.10%	0%	$7.6\times 10^{47}$	+1	$+7.3\times 10^{36}$	
^27^Al	p	1.30%	10.62%	0.45%	53.92%	0%	9.32%	9.53%	0%	$1.9\times 10^{48}$	+1	$+5.6\times 10^{37}$	
^23^Na	p	0%	94.85%	0%	0%	0%	0%	0%	0%	$2.6\times 10^{47}$	−3/5	$-3.4\times 10^{36}$	
^39^K	p	72.50%	0%	20.10%	0%	0%	0%	0%	0%	$1.3\times 10^{46}$	−3/5	$-2.9\times 10^{34}$	

We obtain the spatial distribution of isotope densities $\rho_{i}\left(r\right)$ by integrating geochemical, geophysical, and petrological data. This includes seismic analyses (*49*), geochemical data (*51*), and high-pressure mineral physical experiments (*50*, *52*), which collectively delineate Earth’s structure into crust, mantle, and core. Our focus lies on the crust and mantle, as the core contains negligible concentrations of nonzero-spin isotopes beyond trace amounts of ^57^Fe. Using a representative pyrolite compositional model, we calculate mass fractions of major minerals and rocks as a function of depth and derive elemental densities accordingly. For example, aluminum as the primary source of proton spins is predominantly hosted by plagioclase-(Na,Ca)[(Si,Al)AiSi_2_]O_8_ in the crust, garnet-(Mg,Fe,Ca)_3_Al_2_Si_3_O_12_ in the upper mantle, and bridgmanite-(Mg,Fe)(Si,Al)O_3_ in the lower mantle, as shown in Table 1. The key isotope densities are further refined using natural isotopic abundances, confirming that geoprotons originate mainly from ^27^Al and geoneutrons from ^25^Mg, as shown in Fig. 1B.

We further obtain the nuclear spin polarization based on geomagnetism. Nuclear spin polarization is governed by the alignment of nonzero spins $I_{i}$ with the external magnetic field, yielding a polarization $P_{i}\left(r\right)\propto B\left(r\right)/T\left(r\right)$ that depends on field strength B(r) and ambient temperature T(r). As shown in Fig. 1C, the temperature profile as a function of Earth depths can be obtained directly from geotherm data (*53*), while the external magnetic field the atoms experience is complex. Beyond the geomagnetic field $B_{E}\left(r\right)$, nuclei experience an additional local field $B_{\text{loc}}^{i}$ because of hyperfine coupling with unpaired electrons in paramagnetic minerals.

This local field, which scales as $B_{\text{loc}}^{i}\propto AB_{E}\left(r\right)$ with hyperfine coupling constant *A*, can be accurately modeled on the basis of the lattice structure. Fortunately, in weakly paramagnetic mantle minerals, local fields are about two orders of magnitude weaker than the geomagnetic field, leading to the approximation $B\left(r\right)\approx B_{E}\left(r\right)$ (See Materials and Methods for details). Here, we derive the geomagnetic field inside Earth $B_{E}\left(r\right)$ from the World Magnetic Model (*54*).

Combining the isotopic densities, effective net nucleon numbers, and nuclear spin polarization, we obtain a global map of polarized proton/neutron spin density and orientation, expressed as (see Materials and Methods for details)

$$\rho_{n/p}\left(r\right)=\sum_{i=\text{isotope}}\left[2I_{i}\sigma_{n/p}^{i}\cdot \rho_{i}\left(r\right)\cdot \;\frac{g_{i}\mu_{N}B\left(r\right)\left(\sum_{-I_{i}}^{I_{i}}m^{2}\right)}{I_{i}\left(2I_{i}+1\right)k_{B}\;T\left(r\right)}\right]$$ (4)

where $g_{i}$ is the nuclear *g*-factor of isotope *i*, $k_{B}$ is Boltzmann’s constant, $\mu_{N}$ is the nuclear magneton, and the spin polarization direction aligns with the local geomagnetic field. Figure 1 (D and E) shows the polarized nucleon density sampled directly beneath the surface-NMR detector, aligned with Earth’s rotation axis. We calculate the total number of polarized geoprotons and geoneutrons as $5.3\times 10^{37}$ and $2.2\times 10^{37}$, respectively, with the relevant parameters detailed in Table 1. It is worth noting that these values are provided only to give an intuitive sense of the total number of polarized nucleons within Earth but are not used in the calculation of the exotic interactions. In the Supplementary Materials, we discuss in detail how the effective number of polarized protons varies with the force range.

We further consider the uncertainty in the polarized geonucleon density. This uncertainty arises primarily from uncertainties in the geophysical abundance model (*15*, *51*) and the nuclear structure model (*55*). In our calculation, these sources contribute uncertainties of 6.5 and 11.4%, respectively, leading to a combined uncertainty of 13.1% in $\rho_{n/p}\left(r\right)$. However, the uncertainty in $\rho_{n/p}\left(r\right)$ ultimately contributes less than 1% to the total systematic uncertainty in the final limits and is therefore negligible (see Materials and Methods for details).

### Phase-cycle surface-NMR experiments

We develop a surface-NMR detector integrated with a phase cycling technique for the challenging geonucleon-induced SSI searches. Existing laboratory-scale searches for exotic interactions typically modulate the position or polarization of spin/mass sources to generate oscillatory signals at specific frequencies. For example, in SSI search experiments, spin-based amplifiers require 10-Hz square-wave modulation of a polarized Rb-atom spin source to match the detector’s sensitive band (*30*, *31*), while spin exchange relaxation–free magnetometers need 5.25-Hz rotation of SmCo_5_ sources to avoid the rapidly increasing low-frequency noise (*34*). However, using Earth as a nuclear spin source in the present work renders such artificial modulation impractical, resulting in static exotic fields. The nonmodulated nature of these signals poses fundamental detection challenges for existing detectors, which are generally designed for modulated signal detection rather than absolute field measurements. To address this, we develop a surface-NMR detector based on a ^129^Xe–^131^Xe-Rb system, wherein the two isotopes have opposite gyromagnetic ratios, enabling effective cancelation of common-mode magnetic noise errors. Moreover, a phase cycling technique is used by periodically reversing the bias field and platform tilt. This approach neutralizes noncommon-mode systematic error by two orders of magnitude, thereby enabling absolute measurements of the static exotic signals.

**Fig. 2.**
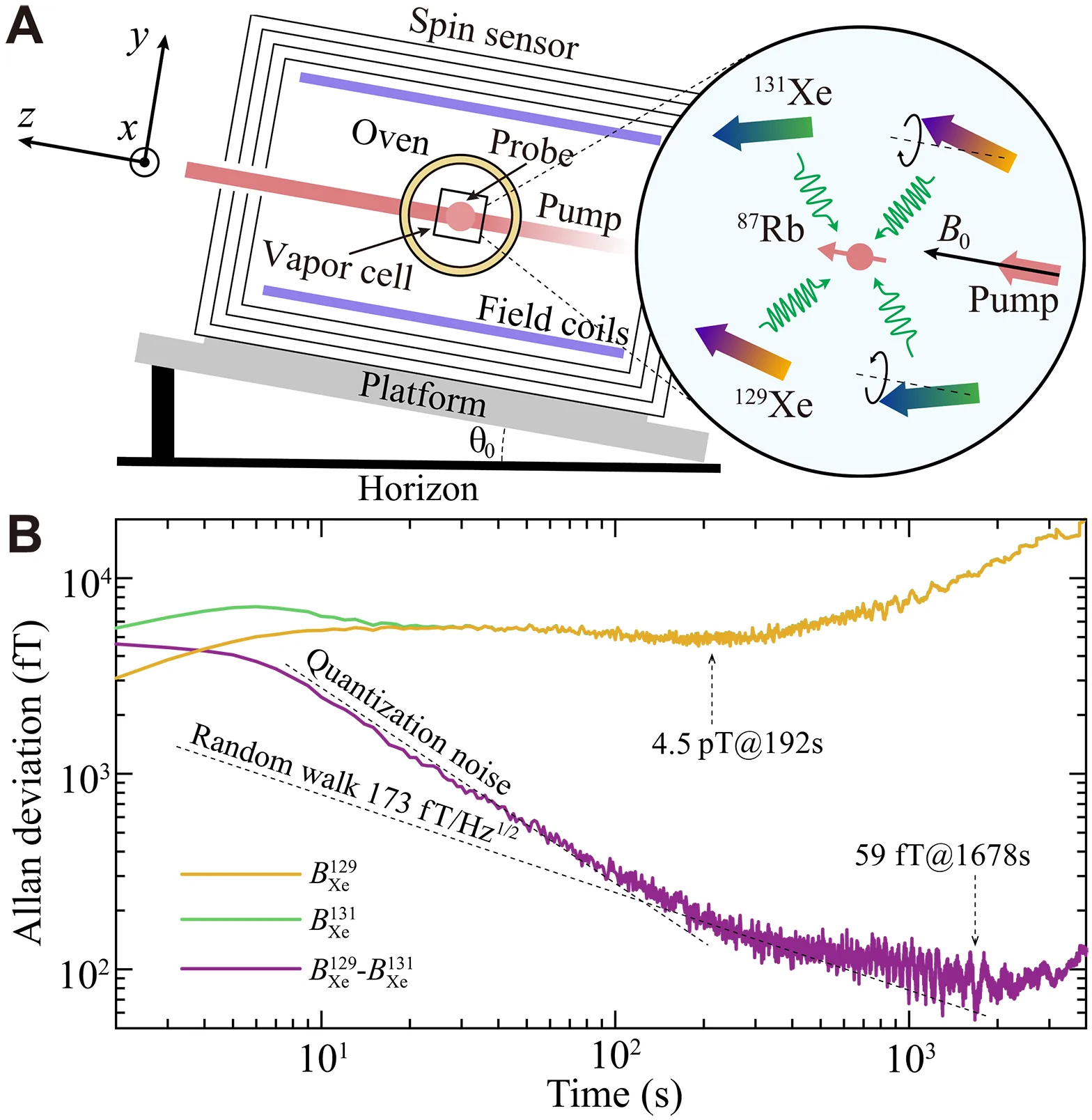
Surface-NMR detector. (**A**) Illustration of experimental apparatus. The detector is mounted on a platform that makes an angle of $\pm 4.5^{\circ }$ to the horizontal. The bias field and the pump light are aligned parallel to the platform, both coinciding with the sensitivity axis. The two xenon isotopes have distinct gyromagnetic ratios with opposing signs, and their precession frequencies differ by about 3.4-fold. Their resonant precession signals are both read out by in situ Rb atoms. (**B**) Allan deviation of detector signals. The Allan deviations of the individual ^129^Xe and ^131^Xe channel outputs, denoted as $B_{\text{Xe}}^{129}$ (green) and $B_{\text{Xe}}^{131}$ (blue), are shown together with that of their differential signal $B_{\text{Xe}}^{129}-B_{\text{Xe}}^{131}$ (purple), which serves as the final detector output. The stabilization time of the surface-NMR system is determined to be 1678 s, corresponding to the bottoming time of the differential-output Allan deviation. The sensitivity at this bottoming point is 59 fT, defining the measurement precision achievable in a single search cycle.

Our surface-NMR detector combs long-coherence nuclear spins from ^129^Xe (I=1/2) and ^131^Xe (I=3/2) with optically polarized Rb atoms, as shown in Fig. 2A. Both xenon isotopes are longitudinally polarized via spin exchange with Rb, and their transverse nuclear spin evolutions are detected in situ through the Fermi-contact interaction with Rb. The apparatus operates under a bias field $B_{0}\hat{z}$ and uses two transverse oscillatory fields precisely matched to the NMR frequency of each isotope. Taking advantage of closed-loop frequency adjustments detailed in Materials and Methods, the detector consistently maintains its most sensitive operating state, allowing for prolonged and stable detection of magnetic, pseudomagnetic, and inertial effects. The output signals from each xenon isotope can be expressed as

$$B_{\text{Xe}}^{i}=B_{0}+\frac{B_{2}^{\text{SSI}}\sigma_{n}^{i}\text{cos}\phi_{0}}{g_{\text{Xe}}^{i}∥S_{n}∥}+\frac{\hbar \Omega_{E}\text{cos}\phi_{1}}{\mu_{N}g_{\text{Xe}}^{i}}+B_{\text{spu}}^{i}$$ (5)

where i=129,131 respectively denotes the ^129^Xe and ^131^Xe; $∥S_{n}∥=1/2$ represents the valence neutron spin; $\sigma_{n}^{129}=1$ and $\sigma_{n}^{131}=-0.2$ are projections of the valence neutron spin on the xenon nuclear spin; $\Omega_{E}\approx 11.6\;\mu$Hz corresponds to Earth’s rotation frequency; $\phi_{0}$ ($\phi_{1}$) is the misalignment angle of $B_{2}^{\text{SSI}}$ ($\Omega_{E}$) compared with $B_{0}\hat{z}$; and $B_{\text{spu}}^{i}$ accounts for spurious effects such as magnetic gradients that can affect the isotopes differently. Conducting nonmodulated SSI searches individually with ^129^Xe or ^131^Xe is impractical because of the fluctuations in the bias field and spurious field, which limit stabilization times for each isotope to under 192 s, as shown by the yellow and green lines of Allan deviations in Fig. 2B.

The ^129^Xe–^131^Xe NMR detector substantially suppresses field fluctuation and drift effects through common-mode differential, combined with the laser stabilization and phase regulation technique. By subtracting the two xenon isotope signals in Eq. 5, the detector becomes insensitive to the bias field $B_{0}$ and the common-mode part from the spurious field $B_{\text{spu}}^{i}$, while preserving sensitivity to $B_{2}^{\text{SSI}}$ and $\Omega_{E}$. Additional stability is achieved by stabilizing the pump laser’s frequency and power that primarily contribute to systematic perturbations and fine-tuning phase delays of the closed-loop frequency to reduce sensitivity to external variables such as temperature. With $B_{\text{Xe}}^{129}-B_{\text{Xe}}^{131}$ as the output, these techniques extend the detector stabilization time to 1678 s and improve the measurement precision by about two orders of magnitude to 59 fT, as shown by the purple line in Fig. 2B. Here, we use Allan deviation analysis to characterize the noise composition and magnitude across different timescales. Figure 2B shows that the differential signal is dominated by quantization noise below about 100 s and by random walk noise between 100 and 1678 s and exhibits a rising noise floor at longer timescales due to fluctuations and drifts of the pump-laser power. This suggests that a single search cycle should last less than 1678 s to ensure optimal detector stability.

**Fig. 3.**
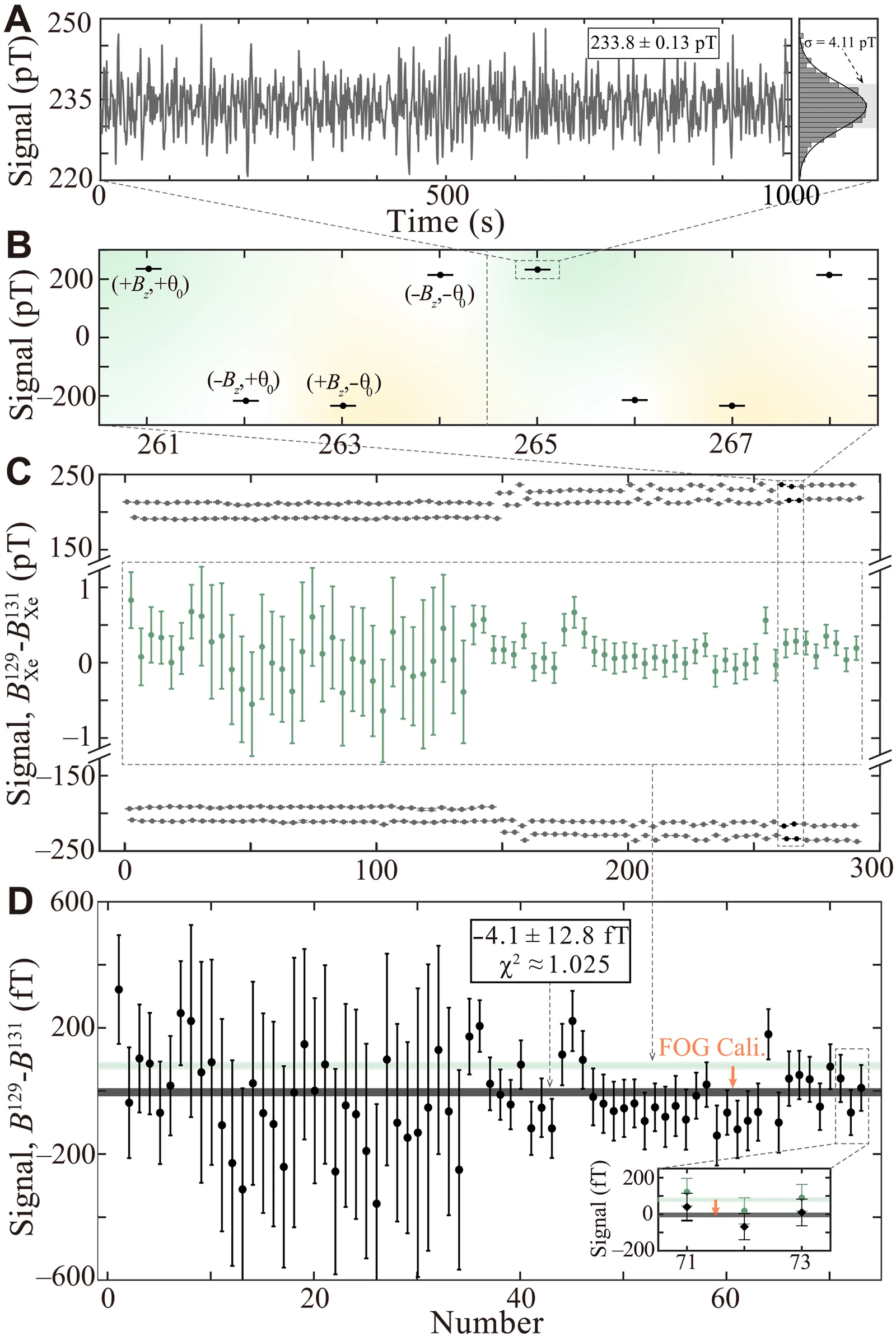
Phase-cycle NMR measurement. (**A**) Measurement of pseudomagnetic fields within a single search cycle by the ^129^Xe–^131^Xe NMR detector. A cycle of 1000 s is shorter than the stabilization time of 1678 s, ensuring the system remains stable during a single search. (**B**) A data bin consisting of four search cycles with different bias fields $\pm B_{0}$ and platform tilts $\pm \theta_{0}$. The uncertainty of each bin comes from the statistical errors of its four contributing data cycles. (**C**) A total of 73 data bins, whose systematic error is reduced to less than 1 pT. (**D**) Illustration of Earth rotation–induced systematic error being eliminated with a fiber-optic gyroscope. The final measured pseudomagnetic field is obtained as −4.1±12.8 fT.

We set each search cycle to 1000 s, obtaining a statistical uncertainty (expressed as SE) of about 130 fT in sensitivity. However, as shown in Fig. 3A, a substantial systematic error in the form of a measurement bias of 233.8 pT remains. This bias, which originates from noncommon-mode parts of $B_{\text{spu}}^{i}$ such as magnetic gradients and the ^131^Xe electric quadrupole moment, exceeds the statistical uncertainty and cannot be removed by simple ^129^Xe–^131^Xe differentiation, thus requiring additional techniques to be effectively suppressed.

To eliminate the systematic error offset, we use four search cycles to alternate the bias field $\pm B_{0}$ and the platform tilt $\pm \theta_{0}$ in turn, as shown in Fig. 3B. This method targets specific SSI signals and neutralizes measurement offset by inverting $B_{\text{spu}}^{i}$, mimicking the traditional NMR phase cycle technique. For example, flipping the bias field directions helps counter magnetic gradient–induced errors, while changing the sensitivity axis tilt compensates for ^131^Xe frequency shifts from long-term temperature drifts. By grouping every four search cycles into a bin, the systematic error can be reduced more than 200-fold to below 1 pT, as shown in Fig. 3C. In addition, a fiber-optic gyroscope linked to the surface-NMR detector corrects for Earth rotation–induced shifts, thereby ensuring that only exotic SSI signals $B_{2}^{\text{SSI}}$ theoretically contribute to the final output. As shown in Fig. 3D, our NMR phase cycle technique suppresses the systematic error of magnetic measurements below the statistical error, allowing for robust exotic SSI searches. Last, a total of 73 such data bins are compiled. Using a weighted chi-square fitting, we obtain a final static field estimate of −4.1 fT, with a measurement precision of 12.8 fT and a chi-square value of $\chi^{2}\approx 1.025$.

### Exotic interaction searches and results

The searches for exotic SSIs are performed over a 1-month period in Hefei, China, at longitude 117.17°E and latitude 31.83°N. The sensitivity axis of the surface-NMR detector is oriented north-south, angled at $\theta_{0}$ relative to the horizontal (Fig. 2A). During the searches, we compile a total of 73×4×1000 s of data, from which the measured pseudomagnetic field is determined to be −4.1±12.8 fT. This result shows the absence of any statistically significant SSI signals in our searches. From this null result, we derive upper limits on the strength of exotic SSI mediated by *Z*′ bosons and axions, specifically $g_{A}^{n}g_{A}^{p}$, $g_{A}^{n}g_{V}^{p}$, and $g_{P}^{n}g_{P}^{p}$—between geoprotons and xenon-isotope neutrons within our detector. As an example, considering a force range of $\lambda =10^{5}$ km, the coupling constant for ${V_{2}+V_{3}|}_{Z^{\prime }}$ is constrained to $g_{A}^{n}g_{A}^{p}\approx \left(-3.4\pm 10.6_{\text{stat}}\pm 4.4_{\text{syst}}\right)\times 10^{-47}$, whose systematic uncertainty $4.4\times 10^{-47}$ aggregates all the residual experimental errors, as detailed in Materials and Methods. This leads to an upper limit of $g_{A}^{n}g_{A}^{p}<2.6\times 10^{-46}$ at 95% confidence level. We extend such constraints across the entire force range by varying λ, as presented in the following analysis.

**Fig. 4.**
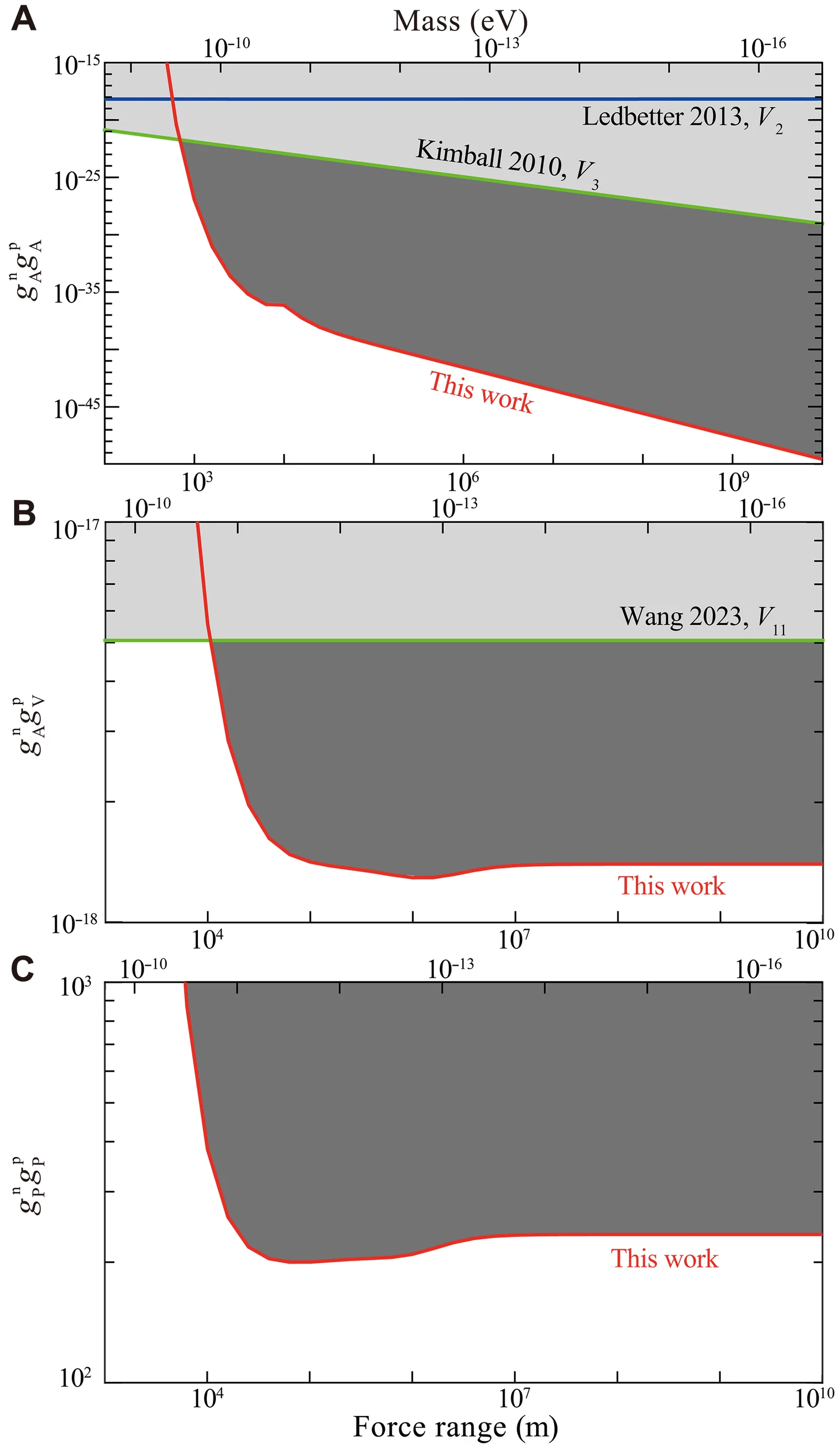
Constraints on spin-spin-interaction coupling constants. The red lines represent our experimental limits on proton-neutron coupling at the 95% confidence level as a function of the force range λ. In our work, the protons are from the Earth source and neutrons from xenon isotopes in the detector. (**A** and **B**) Constraints on the strength of the *Z*′ boson–mediated coupling $g_{A}^{n}g_{A}^{p}$ and $g_{A}^{n}g_{V}^{p}$, respectively. In (A), the blue and green lines show the existing constraints derived from atomic-scale tests of $V_{2}$ and $V_{3}$ (*14*, *56*), respectively. In (B), the green line is from (*31*) where an ^87^Rb vapor cell serves as the proton spin source. (**C**) Constraints on axion-mediated pseudoscalar/pseudoscalar coupling $g_{P}^{n}g_{P}^{p}$.

Figure 4 shows the experimental constraints on SSI strengths set by this work. Previous limits on the proton-neutron axial-vector/axial-vector coupling $g_{A}^{n}g_{A}^{p}$ are based on atomic-scale experiments of ∼1 Å. These include high-precision measurements of spin-exchange cross sections in sodium-helium collisions (*14*) and of NMR *J*-coupling in deuterated molecular hydrogen (*56*), which are both compared against corresponding theoretical predictions to yield SSI strength limits. While such atomic-scale SSI searches achieve high sensitivity at ultrashort force ranges, the fewer spin-source particles they focus on result in substantially lower sensitivity compared with our approach at Earth-scale force ranges, as shown in Fig. 4A. Our Earth-scale surface-NMR searches surpass previous constraints for force ranges λ>0.7 km, improving prior limits (*14*) by more than 15 orders of magnitude at λ=4.0 km (reaching $g_{A}^{n}g_{A}^{p}\approx 2.2\times 10^{-39}$) and beyond. Moreover, our work sets the most stringent constraints on axial-vector/vector coupling $g_{A}^{n}g_{V}^{p}$ for λ>10.7 km, as shown in Fig. 4B. This enhanced sensitivity originates from the vastly greater number of polarized geoprotons involved in our setup. The previous experiment (*31*) using valence protons in ^87^Rb vapor cells contains about $2\times 10^{23}$ fewer polarized protons than Earth.

We also constrain the axion-mediated SSI $V_{3}$, the only interaction directly sensitive to pseudoscalar/pseudoscalar coupling $g_{P}^{n}g_{P}^{p}$. As shown in Fig. 4C, our experimental results extend into previously unexplored parameter space for force ranges beyond 10^3^ m. Previous constraints on $g_{P}^{n}g_{P}^{p}$ coupling between protons and neutrons are restricted to atomic scales of $\lambda <10^{-6}$ m (*14*, *56*) and degrade substantially for force ranges exceeding 1 μm, hence not displayed in Fig. 4C. This absence of large-scale constraints for $g_{P}^{n}g_{P}^{p}$ is attributable to the previous lack of a suitable macroscopic proton spin source—a gap now filled by our innovative use of Earth’s polarized protons.

## DISCUSSION

The construction of the distribution map of Earth’s polarized nuclear spins opens a new pathway for future studies of nucleon-related fundamental physics at macroscopic scales. This geonucleon map enables a wide variety of existing high-sensitivity spin sensors to conduct complementary searches to our work, offer improved sensor sensitivity, or probe different types of exotic interactions. For example, conventional liquid- and solid-state NMR sensors, including those using parahydrogen-induced hyperpolarization (*40*, *41*), use proton-type nuclear spins such as ^1^H, ^19^F, ^7^Li, ^23^Na, and ^31^P as their working nuclei (*42*, *57*–*62*). These systems, which typically achieve a magnetic sensitivity of 120 $\text{fT}/{\text{Hz}}^{1/2}$ (*62*), are naturally suited to investigate proton-proton SSIs, complementing our neutron-proton coupling results. For a measurement time of 1 day, these systems could constrain $g_{A}^{p}g_{V}^{p}=1.9\times 10^{-20}$ at the force range $\lambda =10^{5}$ km, thereby filling a current gap in the study of this coupling (*10*, *63*). Moreover, a growing number of space-deployed quantum sensors, such as in-orbit atomic clocks and interferometers (*64*, *65*), offer a unique opportunity to study exotic spin-dependent interactions originating from Earth. The orbital modulation of space sensors relative to Earth not only naturally modulates the exotic fields to enhance signal extraction but also enables the study of exotic spin-velocity interactions (SVI) between geonucleons and detectors (*66*, *67*). For example, using our Earth’s polarized proton distribution data, an in-orbit hydrogen maser with 0.7-mHz frequency stability (*68*) could probe previously unexplored proton-proton SVIs, potentially constraining the coupling strength of $V_{8}$ (one of the SVIs) to below 10^−11^.

Overall, combining the newly developed Earth’s polarized nucleon map with surface-NMR techniques, we conduct a comprehensive search for all three types of SSIs between protons and neutrons, establishing the most stringent limits on the coupling strengths $g_{A}^{n}g_{A}^{p}$, $g_{A}^{n}g_{V}^{p}$, and $g_{P}^{n}g_{P}^{p}$. Beyond Earth, our approach can also be extended to other planetary bodies, where more abundant polarized spins may enable surface-NMR experiments with substantially enhanced sensitivity. For example, Jupiter—with a mass roughly 300 times that of Earth—contains vast quantities of polarizable ortho-hydrogen molecules in its interior (*69*). Under Jupiter’s magnetic field, which exceeds that of Earth by two orders of magnitude (*70*), these molecules can acquire substantial polarization. We further construct a quantitative model of polarized protons in Jupiter by referring to the density distribution of ortho-hydrogen molecules and the internal temperature profile reported in (*69*) and assuming a representative magnetic field of 0.004 T (*70*) parallel to Jupiter’s rotation axis. According to this model, Jupiter contains $6.6\times 10^{43}$ polarized protons, representing an increase of approximately six orders of magnitude over Earth’s polarized protons estimated in the present work. In this case, the detector should be installed on board a spacecraft orbiting the planet (*66*, *67*). Performing surface-NMR experiments on Jupiter or other similarly massive planets could thus markedly improve sensitivity to exotic spin-dependent interactions and open unexplored windows onto beyond-standard-model physics. The prospect of extending such experimental capabilities from Earth into deep space is becoming increasingly viable, supported by ongoing advances in deep space exploration programs (*71*, *72*) and existing space-based initiatives in quantum communication (*73*) and fundamental physics (*66*, *67*, *74*).

## MATERIALS AND METHODS

### Nuclear spin model

We present how to obtain the valence nucleon spin projection and effective net nucleon number for the target isotope shown in Table 1. Considering isotope *i* with nuclear spin $I_{i}$, its total nuclear spin $I_{i}=S_{n}^{i}+S_{p}^{i}+l_{n}^{i}+l_{p}^{i}$ arises from the contributions of neutron spin $S_{n}^{i}$, proton spin $S_{p}^{i}$, and their respective orbital angular momentum $l_{n}^{i}$ and $l_{p}^{i}$. Since the exotic interactions act upon the nucleon spins rather than the total nuclear spin, it is necessary to confirm the intrinsic spin of protons and neutrons $<S_{n/p}>=\sigma_{n/p}^{i}I_{i}$ for the nucleus, where $\sigma_{n/p}^{i}$ is the neutron/proton spin projection onto the nuclear spin. Further, we can obtain the effective net number of nucleons contained within an atomic nucleus for isotope *i* as

$$\frac{<S_{n/p}>}{S_{n/p}}=2\sigma_{n/p}^{i}I_{i}$$ (6)

In this case, it is crucial to identify the spin projection $\sigma_{n/p}^{i}$ on the basis of the complex nuclear spin model.

We consider the nuclear Schmidt model, a single-particle model used to calculate the magnetic moments of odd nuclei. In this model, the magnetic moment of the nucleus is primarily determined by its unpaired nucleon, i.e., valence neutron or valence proton. The quantum number of nuclear spin and orbital depend on the state of the valence nucleon. For example, the ${}^{39}$K atom comprises 19 protons, with 16 fully occupying the $1s_{1/2}$, $2p_{3/2}$, $2p_{1/2}$, $3d_{5/2}$, and $2s_{1/2}$ levels, and the remaining 3 filling the $3d_{3/2}$ level. Among the three protons, two are coupled into pairs, while the last unpaired proton serves as the valence proton in the $3d_{3/2}$ state (*48*). Therefore, the nuclear spin of ^39^K is I=3/2, and the orbital quantum number is $l_{p}=2$.

On the basis of the Schmidt model, the total nuclear spin can be simply expressed as $I_{i}=S_{n/p}^{i}+l_{n/p}^{i}$. The valence neutron spin projection can be calculated as

$$\sigma_{n/p}^{i}=\frac{<S_{n/p}^{i}\cdot I_{i}>}{I_{i}\left(I_{i}+1\right)}$$ (7)

$$=\frac{I_{i}\left(I_{i}+1\right)+S_{n/p}^{i}\left(S_{n/p}^{i}+1\right)-l_{n/p}^{i}\left(l_{n/p}^{i}+1\right)}{2I_{i}\left(I_{i}+1\right)}$$ (8)

For the ^39^K nucleus, the proton spin projection is calculated as $\sigma_{p}=-1/5$, and the corresponding net nucleon number is $2I\sigma_{p}=-3/5$. We present the calculated results for all key isotopes within Earth in table S1 of the Supplementary Materials.

It is worth noting that there are other nuclear models in addition to the Schmidt model, such as semiempirical EV models (*75*), the minimal, preferred semiempirical FT models (*76*), and full-scale shell model calculations (*77*). Other models yield differing predictions for the nucleon spin projection, and these predictions may not only depend on the valence nucleon (*55*). For example, for ^39^K nuclei in the minimal FT model, the proton spin projection is predicted to be $\sigma_{p}\approx -0.181$, and the neutron spin projection is nonzero as $\sigma_{n}\approx -0.019$. For different models, the predictions for the valence nucleon spin content are reliable to 10%, but the contribution of the nonvalence nucleon spins is very hard to determine even within an order of magnitude (*55*). Therefore, in this work, we adopt the Schmidt model, which is the most widely accepted and extensively used in previous studies, to evaluate nuclear spin characteristics.

### Analysis for magnetic field experienced by nuclei

For a nucleus in Earth materials, the total magnetic field comprises two principal components: the external macroscopic geomagnetic field $B_{E}$, and a microscopic local field $B_{\text{loc}}^{i}$. Given the predominantly paramagnetic nature of mantle minerals and crust rocks, the local field originates mainly from the hyperfine interaction between the nuclear spin and the spins of electrons surrounding the isotope *i* nucleus. The Hamiltonian of the target nucleus can be written as

$$H=H_{\text{Zeeman}}+H_{\text{hf}}=-\gamma_{n}\hbar I_{i}\cdot B_{E}+I_{i}\cdot \left(A\cdot S\right)$$ (9)

where $\gamma_{n}=g_{i}\mu_{N}/\hbar$ is the gyromagnetic ratio of the isotope *i* nucleus; $I_{i}$ is the nuclear spin of the isotope *i* atom; S is electron spins of the surrounding paramagnetic ions; and A is hyperfine coupling tensor.

In paramagnetic systems, the electrons relax much faster than nuclei, enabling the nucleus to perceive the averaged electron spin polarization $\langle S_{z}\rangle$, with $B_{E}=B_{E}\hat{z}$ assumed along the *z*-direction. In this case, the total magnetic field $B=B\hat{z}$ experienced by the nuclear spin $I_{i}$ is expressed as

$$\begin{array}{cc} B & =B_{E}+B_{\text{loc}}\\ & =B_{E}-\frac{A\langle S_{z}\rangle }{\gamma_{n}\hbar } \end{array}$$ (10)

where $B_{\text{loc}}=-A\langle S_{z}\rangle /\gamma_{n}\hbar$ denotes the effective local magnetic field generated by the averaged hyperfine coupling acting on the nucleus. Here, we assume the hyperfine coupling tensor to be isotropic, i.e., A≈A1, where the Fermi contact term dominates and the anisotropy of the dipole term is neglected. The thermally averaged electron spin $\langle S_{z}\rangle$ in paramagnetic systems, using the Brillouin function and its high-temperature approximation, can be derived as (*78*)

$$\langle S_{z}\rangle \approx -\frac{\gamma_{e}\hbar S\left(S+1\right)B_{E}}{3k_{B}T}$$ (11)

where $\gamma_{e}\approx 28$ Hz/nT is gyromagnetic ratio of free electrons and *T* is the local temperature.

Under the geomagnetic field $B_{E}$, the electron spins of paramagnetic ions become partially aligned with the field. Therefore, the effective local magnetic field is

$$B_{\text{loc}}=\frac{A\gamma_{e}S\left(S+1\right)B_{E}}{3\gamma_{n}k_{B}T}$$ (12)

Considering the conditions within Earth’s mantle with T≈2000 K, the ratio of the local field to the external geomagnetic field can be estimated as

$$\frac{B_{\text{loc}}}{B_{E}}\approx 10^{-5}\times \frac{A/h}{\text{MHz}}\times \frac{2000\;K}{T}\times \frac{\gamma_{e}/\gamma_{n}}{10^{3}}\times S\left(S+1\right)$$ (13)

where the hyperfine coupling constant A/h for the key isotopes typically lies in the range of ∼0.1 to 1000 MHz, as shown in Table 2.

**Table 2.** Range of hyperfine coupling constants for key isotopes in Earth mantle and crust.

Isotope	^23^Na	^25^Mg	^27^Al	^29^Si	^39^K	^57^Fe
A/h (MHz)	1–10	2–30	5–30	10–1200	0.5–10	300–800
Ref.	(*84*, *85*)	(*86*, *87*)	(*85*, *88*)	(*85*, *89*)	(*87*)	(*90*, *91*)

We next estimate the magnitude of $B_{\text{loc}}/B_{E}$ by considering two types of paramagnetic minerals used in our study, representing relatively strong and weak paramagnetism. First, we consider the strongly paramagnetic bridgmanite, which contains approximately 10% iron. Assuming that the iron is mainly Fe^2+^, we estimate its effect on the target nucleus ^29^Si. For this case, A/h∼8 MHz (*79*–*81*), $S=2,\;\gamma_{n}=-0.008$ Hz/nT. Therefore, we can acquire the effective local magnetic field

$$B_{\text{loc}}\approx -1.27\times 10^{-3}B_{E}$$ (14)

Next, we consider the weakly paramagnetic plagioclase, which contains only about 1% iron. The iron in this mineral is mainly Fe^3+^, and we estimate its effect on the target nucleus ^23^Na. For this case, A/h∼0.5 MHz (*82*, *83*) $,\;S=5/2,\;\gamma_{n}=0.011$ Hz/nT. The effective local magnetic field is

$$B_{\text{loc}}\approx 8.69\times 10^{-5}B_{E}$$ (15)

In both examples of highly and weakly paramagnetic minerals, the calculated $B_{\text{loc}}$ is negligible compared with the geomagnetic field $B_{E}$. This indicates that $B_{\text{loc}}$ can be safely neglected in our calculations.

### Simulations of Earth-induced exotic interactions

This section presents the simulations of Earth-induced exotic interactions, focusing on the polarized-spin distribution of global geonucleons and the direction of numerically simulated pseudomagnetic fields.

#### Polarized-spin distribution of global geonucleon

We introduce the calculation of the density and polarization direction of polarized geonucleon spins within Earth, $\rho_{n/p}\left(r\right)$. As discussed in the section on polarized geonucleon density map, the polarized geonucleon spin density can be derived from the isotopic number densities $\rho_{i}\left(r\right)$, the effective net nucleon numbers $2I_{i}\sigma_{n/p}^{i}$, and the nuclear spin polarization vectors $P_{i}\left(r\right)$, according to $\rho_{n/p}\left(r\right)=\sum_{i}\left[2I_{i}\sigma_{n/p}^{i}\cdot \rho_{i}\left(r\right)\cdot P_{i}\left(r\right)\right]$. Here, we detail the calculation of isotopic number densities $\rho_{i}\left(r\right)$ and the nuclear spin polarization $P_{i}\left(r\right)$, which together enable the simulation of the polarized spin distribution $\rho_{n/p}\left(r\right)$.

In this work, we focus on isotopes within Earth having polarizable nuclei (i.e., with nonzero nuclear spin), including ^57^Fe, ^25^Mg, ^29^Si, ^27^Al, ^23^Na, and ^39^K. These key isotopes are primarily hosted by various minerals and rocks in the crust and mantle. We consider 17 major mineral species and calculate the contribution of each mineral *s* to the number density of a given isotope *i*, denoted as $\rho_{i}^{s}\left(r\right)$. The total isotope number densities can be estimated by summing over all mineral contributions, $\rho_{i}\left(r\right)\approx \sum_{s}\rho_{i}^{s}\left(r\right)$. The isotope number density $\rho_{i}^{s}\left(r\right)$ contributed by mineral *s* at position r is given by $\rho_{i}^{s}\left(r\right)=\rho_{i,s}^{\prime }\cdot \phi_{s}\left(r\right)$, where $\rho_{i,s}^{\prime }$ is the intrinsic number density of isotope *i* in mineral *s* determined by its crystal structure and isotopic abundance; and $\phi_{s}\left(r\right)$ is the spatial volumetric fraction of mineral *s*. Further, the polarized nucleon density of isotope *i* contributed by mineral *s* can be obtained as $\rho_{n/p}^{i,s}\left(r\right)=2I_{i}\sigma_{n/p}^{i}\;\rho_{i}^{s}\left(r\right)\;P_{i}\left(r\right)$ Integrating $\rho_{n/p}^{i,s}\left(r\right)$ over Earth, we obtain the total number of polarized nucleons hosted by isotope *i* in mineral *s*, $N_{n/p}^{i,s}=\int_{V}\rho_{n/p}^{i,s}\left(r\right)\;\mathrm{dv}$. Last, the polarized spin fraction of isotope *i* contributed by mineral *s* is obtained as $N_{n/p}^{i,s}/\left(\sum_{s}N_{n/p}^{i,s}\right)$, as listed in Table 1. It is worth noting that although we consider 17 mineral species, only 8 minerals that provide the dominant contributions to the polarized spins of a given isotope *i* are presented in Table 1.

**Fig. 5.**
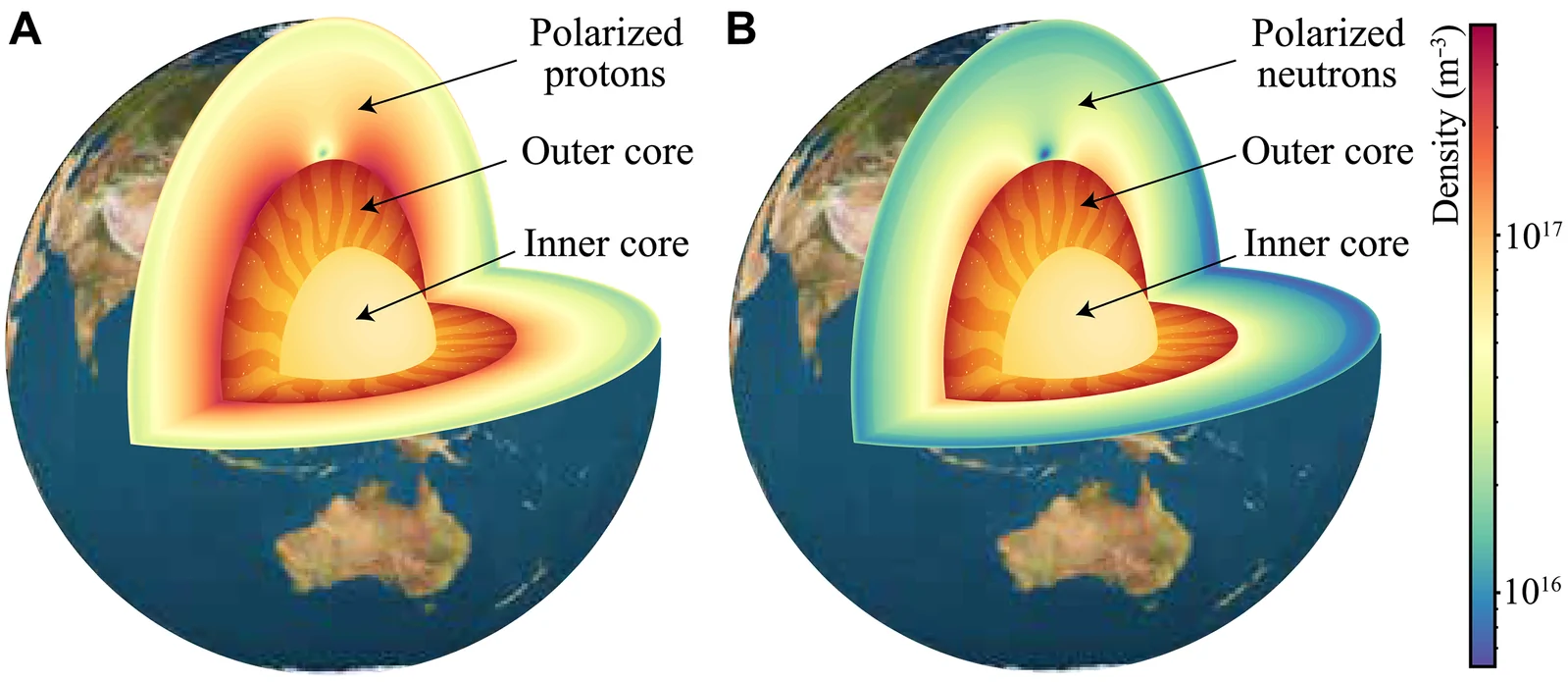
The polarized spin distribution of global geonucleons. (**A**) The polarized proton distribution, which is contributed by ^27^Al, ^23^Na, and ^39^K. (**B**) The polarized neutron distribution, which is contributed by ^57^Fe, ^25^Mg, and ^29^Si.

The spin polarization $P_{i}\left(r\right)$ is another key parameter, characterizing the nuclear polarization degree of isotope *i* at position **r** under an external magnetic field *B* obtained above. Considering an ensemble of identical isotopic nuclei with spin $I_{i}$, the nuclear spin polarization is defined as

$$P_{i}=\frac{\sum_{m}{\mathrm{mN}}_{m}}{I_{i}\sum_{m}N_{m}}$$ (16)

where *m* is the magnetic quantum number and $N_{m}$ denotes the number of nuclei in the state with spin projection *m*. In thermal equilibrium, the nuclei follow the Boltzmann distribution

$$N_{m}=\kappa \;\text{exp}\left[\frac{{\mathrm{mg}}_{i}\mu_{N}B\left(r\right)}{k_{B}\;T\left(r\right)}\right]$$ (17)

where κ is a normalization factor. Inside Earth, the magnetic field B(r) is typically of the order of several tens of microtesla, while the temperature T(r) can exceed 1000 K, satisfying the high-temperature approximation. Therefore, we obtain $\sum_{m}{\mathrm{mN}}_{m}=\frac{\kappa g_{i}\mu_{N}B\left(r\right)}{k_{B}\;T\left(r\right)}\sum_{-I_{i}}^{I_{i}}m^{2}$ and $\sum_{m}N_{m}=\kappa \left(2I_{i}+1\right)$. Substituting these results into Eq. 16, we can lastly acquire the nuclear spin polarization as

$$P_{i}\left(r\right)=\frac{g_{i}\mu_{N}B\left(r\right)\left(\sum_{-I_{i}}^{I_{i}}m^{2}\right)}{I_{i}\left(2I_{i}+1\right)k_{B}\;T\left(r\right)}$$ (18)

where the polarization direction is parallel to the external field direction. As shown in Fig. 5, we lastly determine the polarized spin distribution of global geonucleons following Eq. 4 using the obtained key parameters $2I_{i}\sigma_{n/p}^{i}$, $\rho_{i}\left(r\right)$, and $P_{i}\left(r\right)$.

#### Simulation of pseudomagnetic fields

On the basis of the polarized geonucleon densities $\rho_{n/p}\left(r\right)$ obtained above, we are able to numerically simulate the pseudomagnetic fields induced by exotic SSIs between Earth’s polarized protons and the surface-NMR detector (Fig. 1A). Analogous to the Zeeman interaction, the exotic interaction potential can be expressed in the form $V_{j}=\mu_{N}\;{\hat{\sigma }}_{n}\cdot B_{j}^{\text{SSI}}$. In this case, the phenomenon arising from exotic SSIs can be effectively described as: polarized protons inside Earth generate an exotic magnetic field $B_{j}^{\text{SSI}}$ acting on the surface-NMR detector. Further, the overall pseudomagnetic fields sensed by the NMR sensor can be obtained by integrating the contributions from SSIs throughout Earth

$$\begin{array}{c} B_{3}^{\text{SSI}}=-g_{P}^{n}g_{P}^{p}\int_{V}\rho_{p}\left[{\hat{\sigma }}_{p}\left(\frac{1}{\lambda r^{2}}+\frac{1}{r^{3}}\right)-\hat{r}\right.\\ \left.({\hat{\sigma }}_{p}\cdot \hat{r})\left(\frac{1}{\lambda^{2}r}+\frac{3}{\lambda r^{2}}+\frac{3}{r^{3}}\right)\right]\frac{e^{-r/\lambda }}{16\pi m_{n}^{2}\mu_{N}}dr \end{array}$$ (19)

$$\begin{array}{c} B_{2+3}^{\text{SSI}}=-g_{A}^{n}g_{A}^{p}\int_{V}\rho_{p}\left[{\hat{\sigma }}_{p}\left(\frac{1}{r}+\frac{\lambda }{r^{2}}+\frac{\lambda^{2}}{r^{3}}\right)\right.\\ -\left.\hat{r}\;\left({\hat{\sigma }}_{p}\cdot \hat{r}\right)\left(\frac{1}{r}+\frac{3\lambda }{r^{2}}+\frac{3\lambda^{2}}{r^{3}}\right)\right]\frac{e^{-r/\lambda }}{4{\pi \mu }_{N}}dr \end{array}$$ (20)

$$\begin{array}{c} B_{11}^{\text{SSI}}=-g_{A}^{n}g_{V}^{p}\int_{V}\rho_{p}\left[\left({\hat{\sigma }}_{p}\times \hat{r}\right)\right.\\ \left.\left(\frac{1}{\lambda r}+\frac{1}{r^{2}}\right)\right]\frac{e^{-r/\lambda }}{8\pi m_{n}\mu_{N}}dr \end{array}$$ (21)

where $\rho_{p}\left(r\right)$ is the magnitude of $\rho_{p}\left(r\right)$; ${\hat{\sigma }}_{p}$ is the spin polarization direction, i.e., the direction of $\rho_{p}\left(r\right)$; and *V* represents the integration volume including Earth’s mantle and crust.

In our simulations, the finite element method is used to discretize the integrals in Eqs. 19 to 21 into discrete summations for calculating the pseudomagnetic fields. The polarized protons in Earth’s mantle and crust are discretized into approximately 4.4 million elements. For each element, we calculate the corresponding pseudomagnetic field contribution, and summing over all elements yields the total $B_{j}^{\text{SSI}}$. To ensure numerical accuracy, we confirm that doubling Earth’s subdivision size produces results almost identical to the current ones, demonstrating that the simulation is sufficiently well resolved. Table 3 clearly presents the directions and magnitudes of the pseudomagnetic fields obtained in our simulations, assuming a force range $\lambda =10^{5}$ km and coupling strengths $g_{P}^{n}g_{P}^{p}=10$, $g_{A}^{n}g_{V}^{p}=10^{-19}$, and $g_{A}^{n}g_{A}^{p}=10^{-46}$, corresponding to the parameter space of interest in this work. The results show that the Earth-induced exotic interactions manifest as static pseudomagnetic fields, with notable projections in all three orthogonal directions—west-east direction, north-south direction, and vertical direction.

**Table 3.** Numerical simulations of the pseudomagnetic fields. Results are shown for a force range of $\lambda =10^{5}\;\text{km}$ and coupling strengths $g_{P}^{n}g_{P}^{p}=10$, $g_{A}^{n}g_{V}^{p}=10^{-19}$, and $g_{A}^{n}g_{A}^{p}=10^{-46}$, corresponding to the parameter space of interest in this work.

Pseudomagnetic fields	$B_{3}^{\text{SSI}}$	$B_{2+3}^{\text{SSI}}$	$B_{11}^{\text{SSI}}$
Magnitude	0.21 pT	1.94 pT	0.29 pT
West-east direction	−0.15 pT	−1.37 pT	0.19 pT
North-south direction	−0.12 pT	−1.09 pT	−0.16 pT
Vertical direction	−0.09 pT	−0.85 pT	−0.15 pT

### Contributions of all major elements to the total polarized nucleon density

This section provides a detailed analysis of the contributions of all major elements to the total polarized nucleon density.

We first list all elements in the crust and mantle with a mass fraction greater than 1%, including oxygen, silicon, aluminum, iron, calcium, sodium, potassium, and magnesium, along with their natural isotopes with nonzero nuclear spin, as summarized in table S2 of the Supplementary Materials. In this table, we present our calculations of the total number of polarized nucleon spins contributed by the isotopes of these major elements. Specifically, we first calculate the total number of these isotopes on the basis of their abundances and the corresponding element atom number. Furthermore, on the basis of the effective net nucleon count per atom and the model of Earth’s magnetic field polarization, we calculate the number of polarized nucleon spins contributed by each isotope, as listed in table S2 of the Supplementary Materials.

We now briefly introduce the quantitative contributions of all major elements within Earth to the total polarized nucleon number, as presented in table S2 of the Supplementary Materials . The table shows that Table 1 already includes the dominant isotopic contributions from six major elements, while only oxygen and silicon are not explicitly listed. As a representative example, ^27^Al provides the largest contribution among the included isotopes. Owing to its nearly 100% natural abundance and its relatively high mass fractions in the crust (8%) and mantle (2.5%), ^27^Al contributes the largest polarized nucleon number, reaching $5.6\times 10^{37}$. According to table S2 of the Supplementary Materials, the six isotopes listed in Table 1—^27^Al, ^23^Na, ^39^K (proton spin) and ^25^Mg, ^29^Si, ^57^Fe (neutron spin)—account for more than 99.6% of the total polarized nucleons.

For the elements omitted in Table 1, we also provide quantitative calculations. With oxygen being the most representative example, it is the most abundant element in Earth’s crust and mantle by mass fraction, but its only natural isotope with nonzero nuclear spin, ^17^O, which carries valence neutron spin, constitutes merely 0.039% of all oxygen. Even after integrating over the entire mantle and crust, the total polarized neutron spin contributed by ^17^O is only $3.5\times 10^{35}$, which is less than 2.5% of that contributed by ^25^Mg, the dominant neutron-spin carrier. Similarly, calcium (^43^Ca) also has a natural abundance below 1% and thus contributes negligibly to the total polarized spin budget. Together, the contributions from ^17^O and ^43^Ca amount to less than 0.45% of the total polarized spin number, well below the uncertainty of approximately 10% indicated by the geophysical models. Therefore, their omission from Table 1 does not affect the robustness of our conclusions.

### Uncertainty analysis of the Earth spin-source model

This section presents the calculations of the uncertainties in the polarized geonucleon density, mainly arising from the isotopic number density uncertainties from geophysical models and valence-nucleon projection uncertainties from different nuclear structure models.

We first introduce the geophysical abundance model uncertainties. The complexity of geophysical abundance models leads to uncertainties in the number density of each element at different depths inside Earth, which in turn propagates into the targeted isotope number density $\rho_{i}\left(r\right)$ and, ultimately, into the polarized nucleon density $\rho_{n/p}\left(r\right)$. Table S3 of the Supplementary Materials summarizes our calculations of these uncertainties. These uncertainties primarily originate from three factors (*15*, *51*): uncertainties in the bulk composition of Earth, uncertainties in the mineral composition of the pyrolite model under high-pressure and high-temperature conditions, and deviations in the density-depth profiles inferred from seismic inversions. We calculate the overall uncertainty in the number density of the major elements on the basis of the uncertainties of the three factors, which are ∼1, 3, and 1%, respectively. On the basis of these elemental uncertainties, we propagate them to the isotope uncertainties $\rho_{i}\left(r\right)$ and further through the summation in Eq. 4 to obtain the total uncertainty in the polarized nucleon number. As shown in table S3 of the Supplementary Materials, the overall uncertainty of the total number for polarized geoprotons and geoneutrons due to geophysical abundance models is about $3.4\times 10^{36}$ and $9.6\times 10^{35}$, respectively. This value is used as one component of the total error for polarized spin density. We note that, even under the conservative assumption, the overall uncertainty arising from geophysical abundance models is less than 10% and has a very limited impact, indicating that our calculation of this source of uncertainty is robust.

We further consider the nuclear structure model uncertainties. To quantify this source of uncertainty, we mainly refer to the work in (*55*). The complexity of nuclear structure calculations introduces uncertainty in the nucleon spin projection $\sigma_{n/p}^{i}$, which directly affects the effective net nucleon count per atom $2I_{i}\sigma_{n/p}^{i}$. In our work, we adopt the Schmidt model (a single-particle model) as the standard approach to obtain $\sigma_{n/p}^{i}$, as shown in table S1 of the Supplementary Materials. However, as noted in the section “Nuclear spin model”, there are other nuclear models in addition to the Schmidt model, such as semiempirical EV models, and the minimal, preferred semiempirical FT models. These models yield different predictions for $\sigma_{n/p}^{i}$, and their predictions may not be limited to the valence nucleon alone. For example, for ^39^K in the minimal FT model, the proton spin projection is predicted to be $\sigma_{p}^{{}^{39}K}\approx -0.181$ (compared to the Schmidt model value of $\sigma_{p}^{{}^{39}K}\approx -0.2$), and a nonzero neutron spin projection $\sigma_{n}^{{}^{39}K}\approx -0.019$ also appears, which is absent in the Schmidt model.

To calculate the uncertainty arising from nuclear models, we calculate the polarized nucleon number for each isotope using different nuclear structure models, as shown in table S4 of the Supplementary Materials. As noted in (*55*), the predictions for the valence nucleon spin content are reliable to about 10% or better across different models, whereas the contributions from nonvalence nucleons are very difficult to determine even within an order of magnitude. Therefore, for a conservative estimate, we take the valence-nucleon contributions of Schmidt model as our baseline and take the SD among models as the uncertainty. It is worth noting that we exclude nonvalence nucleon contributions (like neutron in ^39^K) because their uncertainties are too large to be reliably quantified and their contribution is negligible compared with the valence part. As shown in table S4 of the Supplementary Materials, the resulting uncertainty in the total polarized proton/neutron number due to nuclear model variations is about $6.0\times 10^{36}$ and $1.9\times 10^{36}$, respectively.

Combining the geophysical abundance uncertainty and the nuclear model uncertainty in quadrature, we obtain the total uncertainty in the polarized nucleon density. In terms of absolute numbers, the total number of polarized protons in Earth is $\left(5.26\pm 0.69\right)\times 10^{37}$, and the total number of polarized neutrons is $\left(2.16\pm 0.21\right)\times 10^{37}$, corresponding to an associated uncertainty of ∼13.1%. This uncertainty contributes less than 1% to the total systematic uncertainty in the final limits and is therefore negligible.

### Experimental apparatus

**Fig. 6.**
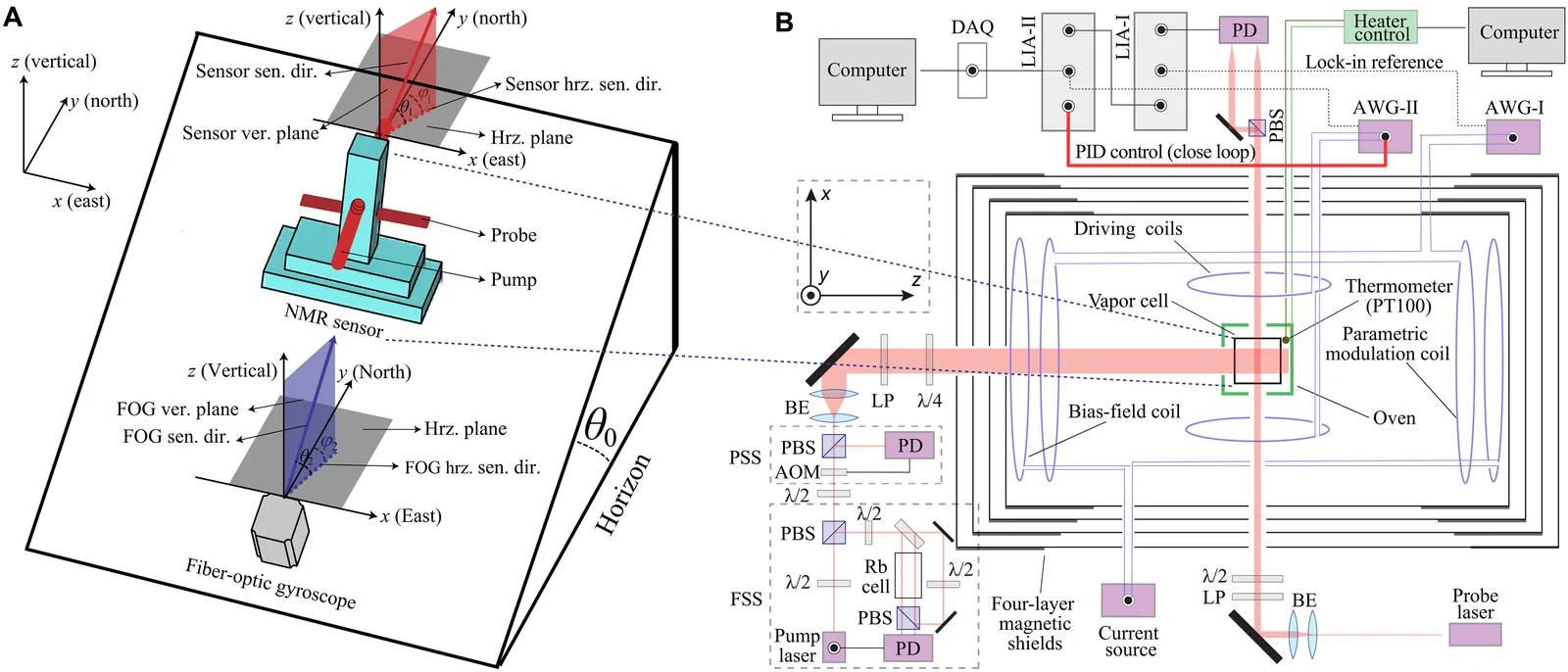
Experimental apparatus. (**A**) Schematics of the experimental apparatus of the detector. For the NMR sensor, only the internal vapor-cell holder, pump, and probe lights are illustrated. The three coordinate axes are defined as along the west-east (*x*), north-south (*y*), and vertical (*z*) directions. We define the *xy* plane as the horizon (hrz.) plane. The plane containing the sensitive (sen.) direction (dir.) and the *z* axis is defined as the vertical (ver.) plane. (**B**) Detailed apparatus of the dual-xenon isotope spin sensor. This schematic adopts a coordinate system different from that in (A): The *z* axis is aligned with the pump beam (sensor sensitive direction), the *x* axis opposite to the probe beam, and the *y* axis orthogonal to both. This convention matches the reference frame described in the main text and Fig. 2A. λ/2, half-wave plate; λ/4, quarter-wave plate; LP, linear polarizer; PBS, polarizing beam splitter; BS, beam splitter; PD, photodiode; DAQ, data acquisition; PSS, power stabilization system; FSS, frequency stabilization system; AWG, arbitrary waveform generator; LIA, lock-in amplifier.

In this section, we provide the details of our experimental apparatus that is used to search for Earth-sourced exotic SSIs. Our detector is located in Hefei, China, at coordinates 31.83°N, 117.13°E. As shown in Fig. 6A, the NMR sensor is mounted alongside a fiber-optic gyroscope (FOG) on a platform inclined at an angle relative to the horizontal plane, with its axis of rotation aligned along the west-east direction. The tilt angle of this platform is adjustable as $\pm \theta_{0}$ to accommodate the phase cycle technique. The FOG used here is single-axis sensitive with a bias stability of about 0.07°/hour.

We carefully calibrate the sensitive directions of the NMR sensor and the FOG. We use a coordinate system where the axes point north-south (longitudinal), west-east (latitudinal), and vertically upward (perpendicular to Earth surface), as shown in Fig. 6A. The sensitive direction of the dual-xenon isotope spin sensor is parallel to its pump beam direction. For each sensor, the inclination angle between its sensitive direction and the horizontal plane is denoted as θ ($\theta_{1}$ for the spin sensor, $\theta_{2}$ for the FOG). Similarly, the azimuthal angle between the horizontal projection of the sensitive direction and the north-south axis is denoted as φ ($\varphi_{1}$ and $\varphi_{2}$, respectively). Each time the platform tilt angle is altered $\pm \theta_{0}$, the parameters $\theta_{1}$, $\theta_{2}$, $\varphi_{1}$, and $\varphi_{2}$ are recalibrated.

We then describe in detail the experimental apparatus of the NMR spin sensor based on two xenon isotopes. As shown in Fig. 6B, the heart of the NMR sensor is a 1 cm by 1 cm by 1 cm cubic vapor cell containing 4-torr isotopically enriched ^129^Xe, 14-torr isotopically enriched ^131^Xe, a droplet of isotopically enriched ^87^Rb, and 450-torr N_2_ as buffer gas. The vapor cell is heated to about 110°C to obtain a sufficient amount of atomic vapor. Here, the gaseous ^87^Rb atoms are polarized along the *z*-direction by a circularly polarized pump laser beam tuned to the D1 transition of Rb. The ^129^Xe and ^131^Xe spins are hereby hyperpolarized by spin-exchange collisions with polarized ^87^Rb spins. The Xe transverse polarizations induced by driving fields are sensed by the embedded Rb atoms and further read out via an *x*-direction linearly polarized probe laser beam, which is detuned 110 GHz to ^87^Rb D2 transition.

A frequency and power stabilization system is implemented in the pump light path to suppress optical noise from frequency and power fluctuations. Frequency stabilization uses saturated absorption spectroscopy to lock the pump light frequency to a specific hyperfine transition of the $D_{1}$ line of ^87^Rb atoms. The frequency-stabilized light then passes through an acousto-optic modulator (AOM), where the 0th-order beam is extracted and split into the primary pump beam and the power-stabilization beam via a polarization beam splitter. The power-stabilization beam is then fed into a PID controller, which maintains stability by continuously adjusting the AOM voltage. Crucially, the constant power ratio between the two beams ensures the simultaneous stabilization of the pump beam’s power.

Three pairs of coils are placed around the vapor cell to generate magnetic fields, including the bias-field coil, the parametric modulation coil, and the driving coil. A permanent bias field $B_{0}$, about 3100 nT, is applied to the atoms along the *z* polarization direction, which yields Larmor frequencies of about 22 kHz for ^87^Rb, 36 Hz for ^129^Xe, and 11 Hz for ^131^Xe. To drive the xenon nuclear spins, an arbitrary waveform generator (AWG-II in Fig. 6B) is used to generate two oscillating fields along the *y*-direction with frequencies (amplitudes) of $\omega_{129}\approx$ 36 Hz (2 nT) and $\omega_{131}\approx$ 11 Hz (3 nT) for ^131^Xe and ^129^Xe, respectively. Moreover, to reduce the low-frequency noise, a parametric modulation field with the frequency (amplitude) of ϕ about 22 kHz (1500 nT) is applied along the *z*-direction via AWG-I to modulate the frequencies of the nuclear spin signals to about 2200±36 Hz and 2200±11 Hz. The aforementioned coils and vapor cell are all enclosed within a four-layer μ-metal shield.

Two lock-in amplifiers (LIAs) are used to obtain the key phase delays $\phi_{\text{Xe}}^{129}$ and $\phi_{\text{Xe}}^{131}$ from the raw nuclear spin signals. These readout phase delays are further fed back to AWG-II, thereby enabling the driving-field frequencies $\omega_{129}$ and $\omega_{131}$ to track the target Larmor frequencies $\Omega_{129}=\gamma_{129}B_{\text{Xe}}^{129}$ and $\Omega_{131}=\gamma_{131}B_{\text{Xe}}^{131}$ described in Eq. 5. This feedback thus forms a close loop for frequency adjustment by keeping phase delays constant.

In specific, the first LIA (LIA-I) is used to demodulate the 22-kHz Rb parametric modulation frequency, thereby extracting the Xe precession signals. The second LIA (LIA-II) further extracts the phase delay from the Xe precession signal

$$\frac{d\phi_{\text{Xe}}^{i}}{dt}=\Delta_{i}-\frac{\text{tan}\left(\phi_{\text{Xe}}^{i}\right)}{T_{2}^{i}}$$ (22)

where i=129,131 respectively denote ^129^Xe and ^131^Xe, $\Delta_{i}=\Omega_{i}-\omega_{i}$ represents the driving field detuning, and $T_{2}^{i}$ is the relaxation time of xenon atoms. The steady-state solution for the phase delay is

$$\phi_{\text{Xe}}^{i}=\text{arctan}\left(\Delta_{i}T_{2}^{i}\right)\approx \Delta_{i}T_{2}^{i}$$ (23)

Therefore, to lock the detuning $\Delta_{i}$ at a constant value, we maintain a constant extracted phase delay $\phi^{i}$. This condition ensures that the driving-field frequency continuously tracks the atomic frequency, thereby keeping the system at its optimal working point. For example, implementing a PID controller to actively adjust $\omega_{i}$ to hold $\phi_{\text{Xe}}^{i}\equiv 0$ directly enforces $\omega_{i}=\Omega_{i}$. Thus, the target field $B_{\text{Xe}}^{i}=\Omega_{i}/\gamma_{i}$ can be directly derived in real time from the AWG-II output frequency $\omega_{i}$.

### Analysis of residual systematic error

This section details the calibration of residual systematic error, including parameter calibration and systematic error calculation. We have described how the mean value of the coupling strength and its statistical error are obtained, whereby the phase cycling technique suppresses the original systematic errors by a factor of 200. Nevertheless, residual systematic errors persist within the system, introduced by calibration uncertainties in key parameters, including the sensor location (longitude and latitude), the sensitive directions of NMR sensor and fiber-optic gyroscope ($\theta_{1}$, $\theta_{2}$, $\varphi_{1}$, and $\varphi_{2}$), and the calculated number of geronucleon, as shown in Table 4. Uncertainties of the locations and the sensitive directions are obtained through the SD of multiple measurements. The uncertainty in the number of polarized geonucleon originates primarily from uncertainties in the geophysical abundance model (*15*, *51*) and the nuclear structure model (*55*). The measured values of the aforementioned parameters and their uncertainties are listed in the second column of Table 4.

**Table 4.** Summary of calibrated parameters and systematic errors. The corrections to $g_{P}^{n}g_{P}^{p}$, $g_{A}^{n}g_{A}^{p}$, and $g_{A}^{n}g_{V}^{p}$ at $\lambda =10^{5}$ km are listed.

Parameter	Calibrated value	$g_{P}^{n}g_{P}^{p}$	$g_{A}^{n}g_{A}^{p}\left(\times 10^{-46}\right)$	$g_{A}^{n}g_{V}^{p}\left(\times 10^{-19}\right)$
Sensor location longitude	$117.13^{\circ }\pm 0.01^{\circ }$ E	<0.001	<0.001	<0.001
Sensor location latitude	$31.83^{\circ }\pm 0.01^{\circ }$ N	<0.001	<0.001	<0.001
NMR sensor sen. dir. $\theta_{1}$	$4.5^{\circ }\pm 1.74^{\circ }$	${}_{-18.0}^{+8.0}$	${}_{-0.20}^{+0.08}$	${}_{-1.08}^{+0.48}$
NMR sensor sen. dir. $\varphi_{1}$	$20.0^{\circ }\pm 1.50^{\circ }$	${}_{-19.6}^{+8.4}$	${}_{-0.22}^{+0.09}$	${}_{+0.60}^{-1.84}$
FOG sen. dir. $\theta_{2}$	$6.45^{\circ }\pm 1.68^{\circ }$	±28.4	±0.31	±1.80
FOG sen. dir. $\varphi_{2}$	$20.69^{\circ }\pm 1.49^{\circ }$	${}_{+0.72}^{-0.16}$	${}_{+0.008}^{-0.003}$	${}_{+0.040}^{-0.012}$
Number of polarized geoproton	$\left(5.26\pm 0.69\right)\times 10^{37}$	${}_{-0.345}^{+0.034}$	${}_{-0.086}^{+0.009}$	${}_{-0.448}^{+0.043}$
Measured value ($\lambda =10^{5}$km)		−30.8±96.4 (stat)	−0.34±1.06 (stat)	−1.76±5.60 (stat)
Overall systematic error		±38.9 (sys)	±0.44 (sys)	±2.83 (sys)

Further, we determine the systematic errors of the coupling couplings caused by the uncertainties of these key parameters. Here, we take the $g_{P}^{n}g_{P}^{p}$ error caused by NMR sensor sensitive direction $\varphi_{1}$ as an example. First, we obtain ${\left(g_{P}^{n}g_{P}^{p}\right)}^{+}$ by using the upper limit of $\varphi_{1}^{+}=20.0^{\circ }+1.50^{\circ }=21.5^{\circ }$ instead of the mean value of the experimental parameter $\varphi_{1}=20.0^{\circ }$ during data analysis. Then, the difference ${\left(g_{P}^{n}g_{P}^{p}\right)}^{+}-g_{P}^{n}g_{P}^{p}\approx +8.4$ is determined as the systematic error caused by the upper limit of $\varphi_{1}$. In the same way, we can determine the systematic error ${\left(g_{P}^{n}g_{P}^{p}\right)}^{-}-g_{P}^{n}g_{P}^{p}\approx -19.6$ caused by the lower limit of $\varphi_{1}$. The systematic errors caused by other key parameters in Table 4 are determined with the same way. Last, we derive the overall systematic error $\pm 38.9_{\text{syst}}$ for $g_{P}^{n}g_{P}^{p}$ by combining all the systematic errors in quadrature.
