# Supplementary material for: Magnetic resonance searches for Earth-sourced exotic nuclear spin–dependent interactions
Source: Sci Adv. 2026 Sep 16;12(38):eaeg0974. doi: 10.1126/sciadv.aeg0974 (PMC13580540; doi:10.1126/sciadv.aeg0974)
Supplement: Supplementary file 1 [file sciadv.aeg0974_sm.pdf]

Science Advances
AAAS

Supplementary Materials for

Magnetic resonance searches for Earth-sourced exotic nuclear spin–dependent interactions

Yuanhong Wang *et al.*

Corresponding author: Min Jiang, dxjm@ustc.edu.cn; Xinhua Peng, xhpeng@ustc.edu.cn

Sci. Adv. **12**, eaeg0974 (2026)
DOI: 10.1126/sciadv.aeg0974

This PDF file includes:

Supplementary Text
Figs. S1 and S2
Tables S1 to S4

I. EFFECTIVE NUMBER OF POLARIZED PROTONS VARYING WITH THE FORCE RANGE

In this section, we provide a detailed discussion of how many polarized protons effectively contribute to the pseudomagnetic signal measured by the detector within a given force range. We would like to clarify that the value of 5.3×10^{37} (denoted by N) in the main text is included only to give readers an intuitive sense of the number of polarized protons distributed within the Earth, but is not used in the calculation of the exotic interactions. In the actual calculation, the pseudomagnetic field $\boldsymbol{B}_2^{\mathrm{SSI}}$ at a given force range λ is obtained by integrating the relevant parameters, such as the polarized geoproton density $\boldsymbol{\rho}_p(\boldsymbol{r}_1)$ and the suppression factor $e^{-r/\lambda}$, over the entire Earth. In the following, we present the detailed calculation procedure for $\boldsymbol{B}_2^{\mathrm{SSI}}$ and provide an intuitive illustration of the relationship between the effective number of polarized protons and the force range λ.

We first briefly describe how the pseudomagnetic field $\boldsymbol{B}_2^{\mathrm{SSI}}$ is calculated in our work. The polarized protons within the Earth are distributed over the vast scale of the entire planet, and for a given force range, protons at different distances can make markedly different contributions. Therefore, for each force range λ, a separate integration over the entire Earth of the relevant parameters, such as $\boldsymbol{\rho}_p(\boldsymbol{r}_1)$ and $e^{-r/\lambda}$, must be performed to obtain $\boldsymbol{B}_2^{\mathrm{SSI}}$. The integral can be written as

$$\boldsymbol{B}_2^{\mathrm{SSI}} = -g_A^n g_A^p \int_0^{2\pi} \int_0^{\pi} \int_{R_{\min}}^{R_{\max}} r_1^2 \sin\theta_1 \frac{\boldsymbol{\rho}_p(\boldsymbol{r}_1) \cdot e^{-r/\lambda}}{4\pi\mu_N r} \, dr_1 \, d\theta_1 \, d\phi_1, \tag{S1}$$

where $\boldsymbol{r}_1$ represents the position vectors of the geoprotons, $\boldsymbol{r}$ denotes the relative position vector between geoprotons and the sensor, μ_N is nuclear magneton, λ is the force range, and $g_A g_A$ is the coupling strength of the axial-vector/axial-vector interaction. The variables r_1, θ_1, and φ_1 are the spherical coordinates of $\boldsymbol{r}_1$, while $R_{\min} = 3476\,\mathrm{km}$ and $R_{\max} = 6371\,\mathrm{km}$ denote the radius of the Earth's core and the Earth, respectively. In the simulations, we employ the finite element method to transform the integral in Eq. (S1) into a discrete summation for calculating the pseudomagnetic field $\boldsymbol{B}_2^{\mathrm{SSI}}$. We first set $dr_1 = 25\,\mathrm{km}$, $d\theta_1 = \pi/160$, and $d\varphi_1 = \pi/120$, thereby discretizing the mantle and crust into 4.4 million elements. For each element, we calculate the corresponding pseudomagnetic field contribution $d\boldsymbol{B}_2^{\mathrm{SSI}}$, and summing over all elements yields the total $\boldsymbol{B}_2^{\mathrm{SSI}}$. Furthermore, to ensure numerical accuracy, we verify that doubling the subdivision size of the Earth yields results nearly identical to the present ones, indicating that our simulation is sufficiently well-resolved.

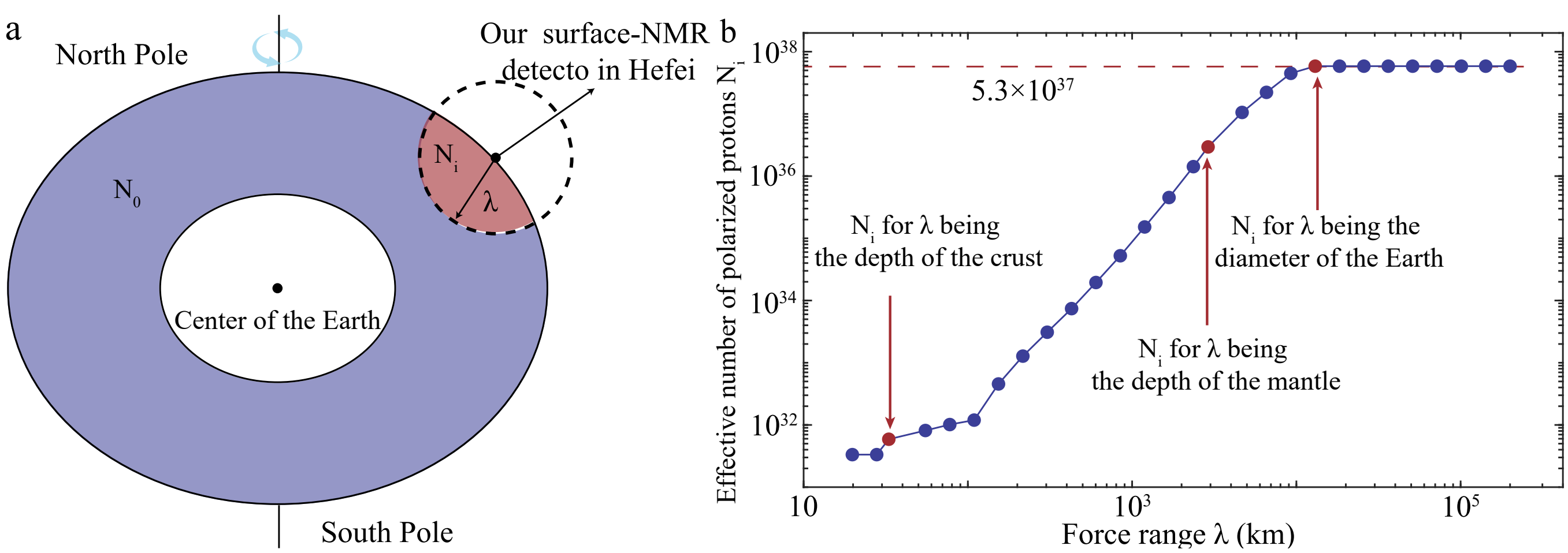

Fig. S1. **Effective number of polarized protons.** (a) Schematic definition of the effective polarized protons. We define the polarized protons located within a distance of one force range from the sensor (the red region in the figure) as the effective polarized protons, whereas those located beyond this distance (the blue region in the figure) are regarded as ineffective polarized protons. Accordingly, the number of polarized protons in the red region, N_i, is defined as the effective number of polarized protons. (b) Dependence of effective number of polarized protons N_i on force range λ. The blue dash-dotted line in the figure shows how N_i varies with λ. In the dash-dotted line, the three red data points represent the effective number of polarized protons at three representative force ranges: 33 km, corresponding to the depth of the crust; 2900 km, corresponding to the depth of the mantle; and 1.3×10^4 km, corresponding to the diameter of the Earth.

We now analyze the relationship between the effective number of polarized protons and the force range λ. To clearly demonstrate this, we adopt a simple approximation in which only particles located within a distance of one force range from the sensor, denoted by N_i, are assumed to contribute, whereas those beyond this distance, denoted by N_o, are assumed to make no contribution, as shown in Fig. S1a. We therefore define N_i as the effective number of polarized protons corresponding to that force range. Based on this definition, we numerically calculate N_i for different force ranges λ, thereby obtaining Fig. S1b below, which provides a simple and intuitive illustration of the dependence of N_i on λ. As shown in Fig. S1b, as the force range increases from 20 km to 200000 km, the effective number of polarized protons gradually increases from approximately 3.0×10^{31} and

eventually converges to 5.3×10^{37}, which is the total number of polarized protons, N, mentioned in the main text. For a force range of 33 km, corresponding to the depth of the crust, N_i is only 5.3×10^{31}, accounting for merely one millionth of the total number of polarized protons, N. When the force range increases to 2900 km, corresponding to the depth of the mantle, this number rises significantly to 2.6×10^{36}, which already amounts to 5% of N. For the force range of 1.3×10^4 km, corresponding to the diameter of the Earth, force range λ is large enough to cover the entire Earth, and N_i has therefore essentially converged to N. Overall, this figure provides an intuitive way to understand how the effective number of polarized protons N_i depends on the force range λ.

II. OPERATIONAL SEQUENCE FOR THE SURFACE NUCLEAR MAGNETIC RESONANCE EXPERIMENT

This section provides a detailed explanation of the ^{129}Xe^{131}Xe operational sequence and the phase-cycling procedure. We first consider the readout signal of the NMR sensor, which can be expressed as

$$\begin{aligned} B_{\mathrm{Xe}} =& A_{\mathrm{SSI}}B^{\mathrm{SSI}}+A_{\Omega_{\mathrm{E}}}\Omega_{\mathrm{E}}+B_{AF}+B_{GF} \\ &+\frac{\hbar}{\mu_N}\left\{\frac{\left(\varphi^{131}-\varphi^{131}_{\mathrm{Rb}}\right)\Gamma_2^{131}}{g^{131}_{\mathrm{Xe}}}-\frac{\left(\varphi^{129}-\varphi^{129}_{\mathrm{Rb}}\right)\Gamma_2^{129}}{g^{129}_{\mathrm{Xe}}}\right\}, \end{aligned} \tag{S2}$$

where $B_{\mathrm{Xe}}=B^{129}_{\mathrm{Xe}}-B^{131}_{\mathrm{Xe}}$, $A_{\mathrm{SSI}}=\frac{\cos\phi_0}{\|S_n\|}\left(\frac{\sigma_n^{129}}{g^{129}_{\mathrm{Xe}}}-\frac{\sigma_n^{131}}{g^{131}_{\mathrm{Xe}}}\right)$, $A_{\Omega_{\mathrm{E}}}=\frac{\hbar\cos\phi_1}{\mu_N}\left(\frac{1}{g^{129}_{\mathrm{Xe}}}-\frac{1}{g^{131}_{\mathrm{Xe}}}\right)$, B_{AF} represents the influence of the alkali field, and B_{GF} represents the effect induced by external magnetic-field gradients through diffusive motion of the Xe atoms. Here, Γ_2^i denotes the transverse spin-relaxation rate of the Xe isotope, φ^i is the phase difference between the response signal to the driving field and the driving field itself, and $\varphi^i_{\mathrm{Rb}}=-\frac{\omega_i}{\Gamma_{\mathrm{Rb}}}$ represents the phase offset introduced by the Rb magnetometer with Γ_{Rb} being the Rb spin-relaxation rate and ω_i being the frequency of the resonant transverse driving field. As can be seen from

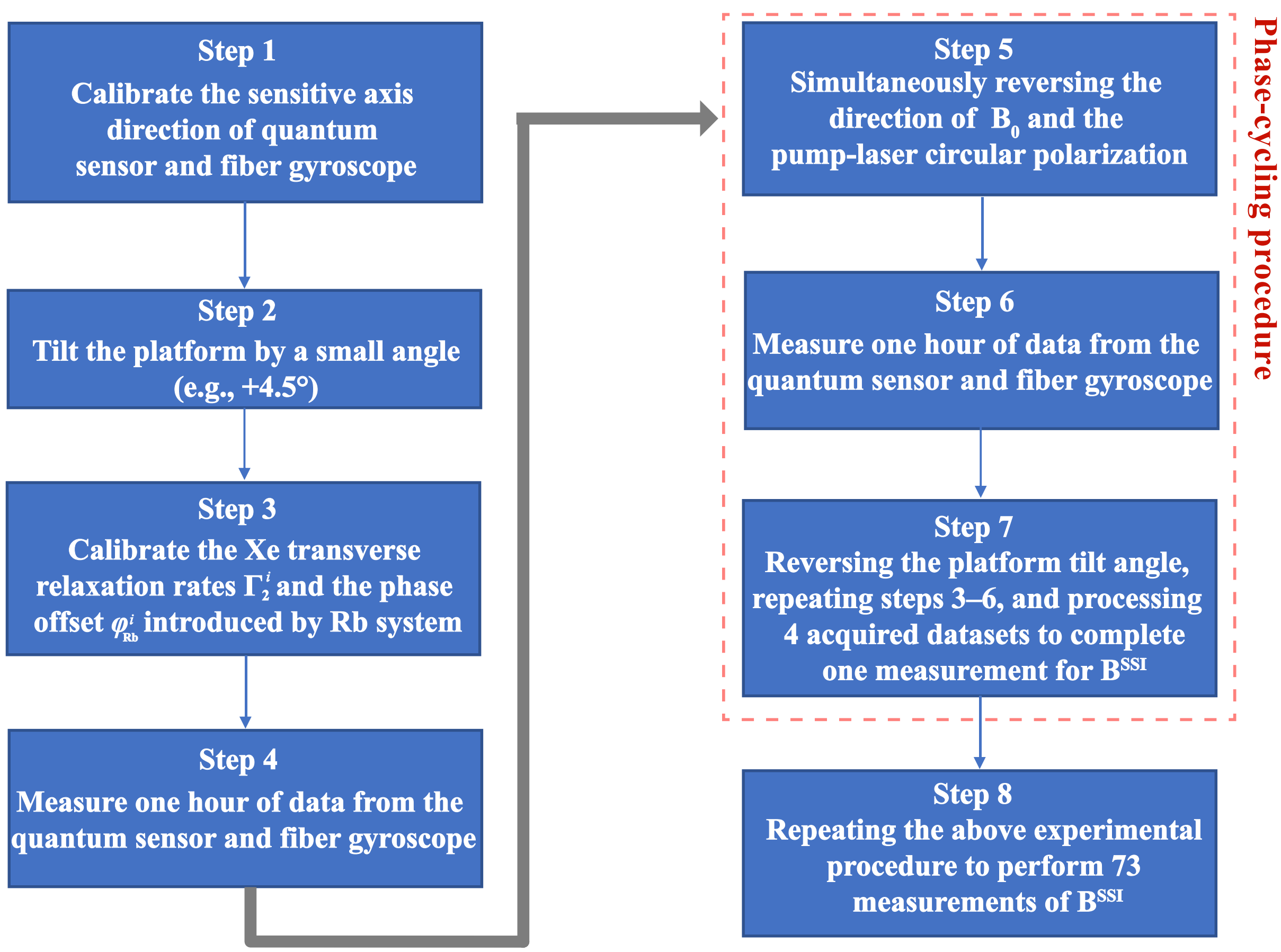

Fig. S2. Operational sequence for the surface nuclear magnetic resonance experiment.

Eq. (S2), the opposite signs of the gyromagnetic ratios prevent the contributions of $B^{\rm SSI}$ to $B^{i}_{\rm Xe}$ from canceling in $B_{\rm Xe}$, thereby yielding a relatively large $A_{\rm SSI}$ and allowing $B^{\rm SSI}$ to be detected by our system. To extract $B^{\rm SSI}$ from the measured signal $B_{\rm Xe}$, several systematic error contributions must be removed, including the gyroscopic effect of Earth's rotation, $A_{\Omega_{\rm E}}\Omega_{\rm E}$, the phase-error term $\frac{\hbar}{\mu_N}\left\{\frac{(\varphi^{131}-\varphi^{131}_{\rm Rb})\Gamma^{131}_2}{g^{131}_{\rm Xe}}-\frac{(\varphi^{129}-\varphi^{129}_{\rm Rb})\Gamma^{129}_2}{g^{129}_{\rm Xe}}\right\}$, alkali-field error B_{AF}, and the error of magnetic-field gradients B_{GF}. Below, following the experimental sequence shown in Fig. S2, we describe in detail how these error terms are eliminated in our experiment.

1. Suppression of the gyroscopic effect: To eliminate the gyroscopic effect associated with Earth's rotation, we first calibrate the relative orientation between the sensitive axes of the quantum sensor and the fiber gyroscope in step 1 of Fig. S2. Then, in steps 4 and 6, the gyroscopic term $A_{\Omega_{\rm E}}\Omega_{\rm E}$ can be determined from the synchronous fiber-gyroscope data together with the calibrated relative orientation obtained in step 1. Subtracting this term from the simultaneously acquired quantum-sensor signal $B_{\rm Xe}$ removes the error introduced by the gyroscopic effect.

2. Suppression of the phase error: To eliminate the phase error, we first need to determine the phase offset in the Xe response, $\varphi^{i}_{\rm Xe}=\varphi^{i}-\varphi^{i}_{\rm Rb}$, as shown in step 3 of Fig. S2. Here, φ^{i} is the lock point of the PID feedback. To improve the stability of the sensor, we employ the optimal-phase method, such that φ^{i} is not zero but instead a fixed value. The term $\varphi^{i}_{\rm Rb}$ denotes the additional phase shift imposed on the Xe precession signal by the Rb magnetometer, which is proportional to the frequency ω_i of the resonant transverse driving field. Therefore, to determine $\varphi^{i}_{\rm Rb}$, we first calibrate the slope of the $\varphi^{i}_{\rm Rb}$-versus-frequency relationship by applying several off-resonant transverse magnetic fields with different frequencies and recording the corresponding phase shifts. Multiplying this calibrated slope by ω_i then gives $\varphi^{i}_{\rm Rb}$, as shown in step 3 of Fig. S2. After calibrating $\varphi^{i}_{\rm Xe}$, we further determine Γ^{i}_2 to evaluate the magnitude of the phase-error term. Finally, this calibrated phase-error term is subtracted from the measured signal $B_{\rm Xe}$, thereby removing the corresponding error.

3. Suppression of B_{AF} and B_{GF}: We now introduce how B_{AF} and B_{GF} are suppressed through the phase-cycling procedure implemented in steps 5-7 of Fig. S2. Here, B_{AF} denotes the alkali-field contribution associated with the polarization of the Rb atoms, whereas B_{GF} denotes the effect induced by external magnetic-field gradients. Accordingly, in step 4, reversing the circular polarization of the pump laser flips the direction of the Rb polarization and thus reverses the sign of B_{AF}. Meanwhile, reversing the direction of the bias magnetic field B_0 changes the sign of B_{GF} arising from the gradient of this dominant field. Notably, reversing the direction of B_0 also reverses the sensor sensitivity axis. Consequently, in the measured signal $B_{\rm Xe}$, the signs of B_{AF} and B_{GF} remain unchanged, while the sign of $B^{\rm SSI}$ is reversed. Therefore, taking the difference between $B^{(1)}_{\rm Xe}$ measured in step 4 and $B^{(2)}_{\rm Xe}$ measured in step 6 can largely suppress the contributions from B_{AF} and B_{GF}. Nevertheless, since the gradient field is not contributed entirely by the bias field B_0, B_{GF} cannot be completely eliminated in this way. To further suppress this residual contribution, in step 7, we tilt the platform in the opposite direction and obtain two additional measurements, $B^{(3)}_{\rm Xe}$ and $B^{(4)}_{\rm Xe}$. We then construct the combination $\left[\left(B^{(1)}_{\rm Xe}-B^{(2)}_{\rm Xe}\right)-\left(B^{(3)}_{\rm Xe}-B^{(4)}_{\rm Xe}\right)\right]/4$, which removes the remaining B_{GF} contribution while retaining the $B^{\rm SSI}$ term due to its contribution differing for the two opposite tilt angles.

III. INNOVATIONS OF THE SURFACE NMR DETECTOR

In this section, we provide a detailed discussion of the innovations of our surface NMR detector. We emphasize that although the NMR detector used in this work is based on a spin system similar to some existing comagnetometers, our sensor differs markedly in measurement scheme and is supported by a comprehensive investigation of various systematic errors. These two points make our sensor extremely suitable for absolute measurements of exotic interactions. Benefiting from its excellent absolute-measurement capability, our NMR detector is able to set the most stringent limits on the three types of exotic interactions investigated in the mian text. Below, we provide a detailed comparison between our NMR detector and other existing comagnetometers from two perspectives: the measurement scheme and the measurement sensitivity.

Before presenting a detailed comparison, we first emphasize that measuring Earth-sourced exotic interactions places strict requirements on sensor design. Unlike previous studies of CPT symmetry and exotic interactions using a modulated spin source to generate AC signals, our work uses the Earth as a non-modulatable source of polarized nucleons, thereby placing stringent requirements on the sensor's ability to measure DC fields. Unfortunately, DC measurements are strongly affected by environmental noise and systematic errors, which necessitates a detailed analysis of systematic effects and makes the experiment considerably more challenging than previous studies involving AC-signal measurements. Following the above criteria, we now present a comparison of three types of comagnetometers that have already been used in searches for exotic interactions: **(1) Dual noble-gas comagnetometers, (2) Hg-based comagnetometers, and (3) alkali-metalnoble-gas comagnetometers.**

(1) Dual noble-gas comagnetometers: Such comagnetometers are typically based on two types of noble gases, such as ^{129}Xe-^{131}Xe and ^{3}He-^{129}Xe. In these comagnetometers, alkali-metal atoms, such as ^{87}Rb, spatially overlap with the noble gases and are used to polarize and read out their nuclear spins. Experimentally, these sensors typically measure the Larmor precession frequencies of the two noble-gas species by applying a transverse pulse to excite free-induction-decay (FID) signals, thereby

enabling the measurement of the pseudomagnetic field generated by exotic interactions. The use of two noble-gas species helps suppress the influence of real magnetic noise and thus improves the sensitivity to the pseudomagnetic field. Among existing studies using this type of comagnetometer for purely DC pseudomagnetic-field measurements of exotic interactions, the highest measurement precision is reported in the work of (*19*), which achieves a magnetic-field sensitivity of $506\,\mathrm{fT/Hz^{1/2}}$.

The main difference between our NMR detector and previous dual-noble-gas comagnetometers is that the measured signals in our system correspond to different solutions of the spin dynamics, which offers distinct advantages in our measurement sensitivity. Specifically, previous dual-noble-gas comagnetometers rely on transient measurements of FID signals, whereas our sensor operates in a closed-loop continuous-detection mode. Experimentally, this closed-loop continuous detection is implemented by continuously applying transverse resonant driving fields to track the Larmor precession frequencies, with the driving frequencies feedback-adjusted according to the phase shifts in the sensor response to the applied fields. From the perspective of spin dynamics, the FID-based measurements used in previous comagnetometers and the closed-loop continuous-detection scheme adopted here correspond to different solutions of the spin dynamics equations: the former corresponds to the transient solution, whereas our case corresponds to the steady-state solution. Since the steady-state solution yields a larger response signal than the transient solution, our detection scheme can achieve higher measurement sensitivity under comparable noise conditions. Specifically, our NMR detector achieves a sensitivity of $173\,\mathrm{fT/Hz^{1/2}}$, representing an approximately threefold improvement over the $506\,\mathrm{fT/Hz^{1/2}}$ reported in the representative dual noble-gas comagnetometer work (*19*).

In addition to the measurement scheme, the systematic errors can also significantly affect the final limits we obtain. For FID-mode comagnetometers, systematic errors have been thoroughly analyzed in previous studies, which is discussed in detail in (*19*). These sensors quantitatively characterize the contributions of various parameters to systematic noise by deliberately varying experimental conditions, and they can also suppress quadrupole-induced errors by combining the three resonance peaks arising from the electric quadrupole splitting of ^{131}Xe. However, in our continuous-operation mode, the analysis of systematic errors becomes more challenging due to additional noise sources introduced by the closed-loop feedback system, such as the phase noise of the Rb magnetometer. Moreover, such sensors have not previously been used to measure a DC pseudomagnetic field, and thus the associated systematic effects of such a sensor were previously unclear. In our work, we present the first use of a closed-loop sensor as a DC pseudomagnetic-field detector, and devote considerable effort to systematically analyzing the systematic errors associated with various physical parameters and developing multiple strategies to suppress them.

We now discuss the analysis and suppression of systematic errors in our work. Many important physical parameters in our experiment, including the laser power P, laser frequency ν, temperature T, and bias magnetic field B_0, can affect the final output of the sensor. Their long-term drifts therefore introduce significant systematic errors. To suppress these parameter-induced systematic errors, we have pursued two complementary strategies: reducing the long-term drifts of the key physical parameters themselves and minimizing their correlations with the final measurement result. To suppress the long-term drifts of the key parameters, we have employed an optical setup equipped with laser-power and laser-frequency stabilization systems to reduce the drifts of P and ν, a specially designed oven together with a closed-loop temperature-control system to stabilize T, and a low-noise current source to suppress the long-term drift of B_0. Here, we describe how the optimal-phase method is used to further reduce the correlations between the measurement result and these parameters. The measured output of our sensor can be written as

$$
\begin{aligned}
B_{\mathrm{Xe}} = & A_{\mathrm{SSI}}B^{\mathrm{SSI}} + A_{\Omega_{\mathrm{E}}}\Omega_{\mathrm{E}} + B_{AF} \\
& + \frac{\hbar}{\mu_N}\left\{\left(\frac{\varphi^{131}\Gamma_2^{131}}{g_{\mathrm{Xe}}^{131}} - \frac{\varphi^{129}\Gamma_2^{129}}{g_{\mathrm{Xe}}^{129}}\right) + \left(\frac{\omega_{131}\Gamma_2^{131}}{g_{\mathrm{Xe}}^{131}} - \frac{\omega_{129}\Gamma_2^{129}}{g_{\mathrm{Xe}}^{129}}\right)/\Gamma_{Rb}\right\},
\end{aligned}
\tag{S3}
$$

where $B_{\mathrm{Xe}} = B_{\mathrm{Xe}}^{129} - B_{\mathrm{Xe}}^{131}$, $A_{\mathrm{SSI}} = \frac{\cos\phi_0}{\|S_n\|}\left(\frac{\sigma_n^{129}}{g_{\mathrm{Xe}}^{129}} - \frac{\sigma_n^{131}}{g_{\mathrm{Xe}}^{131}}\right)$, $A_{\Omega_{\mathrm{E}}} = \frac{\hbar\cos\phi_1}{\mu_N}\left(\frac{1}{g_{\mathrm{Xe}}^{129}} - \frac{1}{g_{\mathrm{Xe}}^{131}}\right)$, and B_{AF} represents the alkali-field influence. Here, Γ_2^i denotes the transverse spin-relaxation rate of the Xe isotope, Γ_{Rb} is the Rb spin-relaxation rate, ω_i is the frequency of the transverse driving field applied resonantly to the Xe isotope, and φ^i is the phase difference between the response signal of our detector to the driving field and the driving field itself. The physical parameters discussed above affect the final measurement result B_{Xe} through their influence on B_{AF}, Γ_2^i, and Γ_{Rb}. According to Eq. (S3), the relationship between B_{Xe} and the possible parameters X can be expressed as:

$$
\begin{aligned}
\frac{\partial B_{\mathrm{Xe}}}{\partial X} = & \frac{\partial B_{AF}}{\partial X} + \frac{\hbar}{g_{\mathrm{Xe}}^{131}\mu_N}\left(\varphi^{131} - \frac{\omega_{131}}{\Gamma_{Rb}}\right)\frac{\partial \Gamma_2^{131}}{\partial X} - \frac{\hbar}{g_{\mathrm{Xe}}^{129}\mu_N}\left(\varphi^{129} - \frac{\omega_{129}}{\Gamma_{Rb}}\right)\frac{\partial \Gamma_2^{129}}{\partial X} \\
& - \frac{\hbar}{(\Gamma_{Rb})^2\mu_N}\left(\frac{\omega_{131}\Gamma_2^{131}}{g_{\mathrm{Xe}}^{131}} - \frac{\omega_{129}\Gamma_2^{129}}{g_{\mathrm{Xe}}^{129}}\right)\frac{\partial \Gamma_{Rb}}{\partial X}.
\end{aligned}
\tag{S4}
$$

The closed-loop phases φ^1 and φ^2 are controllable variables. Thus, theoretically, for any two given parameters, we can always determine their optimal closed-loop phases such that the first-order derivatives of B_{Xe} with respect to the two parameters are simultaneously zero, thereby significantly enhancing the stability of the sensor. Experimentally, by setting the optimal phase, the

noise contributions from the pump laser power P and the vapor cell temperature T to B_{Xe} are substantially reduced. It is worth noting that, in addition to these methods, we also employed the phase-cycling procedure to eliminate the effects of several types of systematic errors. After applying the above methods, the systematic noise contributions from P, ν, T, and B_0 to B_{Xe} were all significantly reduced to below the sensor sensitivity.

(2) Hg-based comagnetometers: Such comagnetometers are typically based on two isotopes of Hg atoms, such as ^{199}Hg-^{201}Hg. Unlike dual-noble-gas comagnetometers, which employ alkali-metal atoms to polarize the noble gases and to detect their precession signals, Hg-based comagnetometers use laser light directly both to polarize and to detect the nuclear spins of Hg atoms. Experimentally, the Hg nuclear spins are first polarized by resonant circularly polarized light into a direction perpendicular to the bias magnetic field, after which they undergo free precession in the bias field. Their FID-like precession signals are then read out using either far-detuned linearly polarized probe light or weak circularly polarized probe light with polarization modulation. The measured free-precession signals are used to determine the Larmor precession frequencies of the Hg atoms, thereby enabling measurement of the pseudomagnetic field arising from exotic interactions. A representative study employing an Hg-based comagnetometer to search for exotic interactions through the measurement of a purely DC pseudomagnetic field is reported in (*38*), in which the measurement sensitivity to the pseudomagnetic field reached approximately $400\,\mathrm{pT/Hz^{1/2}}$.

Our NMR detector differs most significantly from Hg-based comagnetometers in the signal readout mechanism, which provides distinct advantages in measurement sensitivity. Our sensor employs spatially overlapping ^{87}Rb atoms as an in-situ magnetometer to detect the precession signals of Xe atoms, whereas Hg-based comagnetometers directly read out the nuclear-spin signals of Hg atoms optically. In our scheme, the detection of Xe polarization via the ^{87}Rb magnetometer relies on the Fermi-contact interaction between ^{87}Rb and Xe atoms, through which the effective magnetic field exerted by the Xe atoms on the Rb atoms is enhanced by several hundred to several thousand times relative to the magnetic field directly experienced by the Xe atoms. The magnetic-field signal sensed by the Rb atoms is then read out optically using laser light. This gives our detector a major advantage over Hg-based comagnetometers employing direct optical readout in terms of signal response, thereby greatly improving the achievable magnetic-field sensitivity. Specifically, our NMR detector achieves a sensitivity of $173\,\mathrm{fT/Hz^{1/2}}$, which is more than three orders of magnitude better than the $400\,\mathrm{pT/Hz^{1/2}}$ reported in the representative Hg-based comagnetometer work (*38*).

(3) Alkali-metalnoble-gas comagnetometers: Such comagnetometers are typically based on one alkali-metal species and one noble-gas species, such as Rb-^{21}Ne and K-^{3}He. In these comagnetometers, an additional alkali-metal species is first optically pumped, and the working alkali atoms, Rb or K, are then uniformly polarized through alkalialkali spin exchange. The polarized Rb or K atoms subsequently transfer their polarization to the noble-gas spins via alkalinoble-gas spin exchange. At the same time, the alkali-metal atoms serve as in-situ magnetometers that simultaneously detect the external magnetic field and the polarization signal of the noble gas. Experimentally, these devices usually operate in a comagnetometric mode near the compensation point, which makes the system largely insensitive to real magnetic-field noise while maintaining high sensitivity to pseudomagnetic fields arising from exotic interactions. At present, they are mainly used to detect modulated AC pseudomagnetic fields generated by exotic interactions, for which extremely high magnetic-field sensitivity can be achieved. For example, the work reported in (*35*) uses an Rb-^{21}Ne comagnetometer to measure an AC-modulated signal at 0.04 Hz.

Our NMR detector differs markedly from alkali-metalnoble-gas comagnetometers in its measurement scheme. The distinctive measurement strategy of the alkali-metalnoble-gas comagnetometers makes it unsuitable for absolute measurements. In our sensor, the Larmor precession frequencies of the two Xe isotopes are measured directly, so the magnetic field experienced by each isotope can be obtained straightforwardly by dividing the measured frequency by its gyromagnetic ratio. In contrast, alkali-metalnoble-gas comagnetometers operate in the self-compensation point and measure the response of the spin system to the transverse magnetic fields, and thus these fields must then be inferred from the measured voltage signal through an additional calibration procedure. To perform this conversion, an additional calibration is required to determine the voltage-to-field conversion factor. However, this factor depends on several experimental parameters, including the probe laser power, the longitudinal polarization of the alkali-metal atoms, and their relaxation rates, and is therefore time dependent. As a result, a factor obtained from a single calibration may become inaccurate over time, so converting the measured voltage response back into a magnetic field can introduce significant systematic noise, which is unfavorable for absolute measurements. In addition, alkali-metalnoble-gas comagnetometers rely on the environmentally sensitive electron spins of alkali-metal atoms, and even slight deviations from the self-compensation point can severely affect the measurement signal, thereby leading to larger systematic errors and greater long-term drift, further limiting their suitability for absolute measurements. For these reasons, existing alkali-metalnoble-gas comagnetometers have mainly been used in experiments where the pseudomagnetic signal is converted into an AC signal by periodically modulating the polarized source, rather than in direct absolute measurements.

Table S1. **Characteristics of nuclear spin for the key isotopes in the Schmidt model.** The nuclear spin type depends on being an odd-proton nucleus or an odd-neutron nucleus. The valence nucleon spin projection is calculated following Eq. (8) in the main text.

Isotope	Proton number	Neutron number	Spin type	I_i	$\sigma^i_{n/p}$	$2I_i\sigma^i_{n/p}$
^{23}Na	11	12	p	3/2	-1/5	-3/5
^{25}Mg	12	13	n	5/2	1/5	+1
^{27}Al	13	14	p	5/2	1/5	+1
^{29}Si	14	15	n	1/2	+1	+1
^{39}K	19	20	p	3/2	-1/5	-3/5
^{57}Fe	26	31	n	1/2	-1/3	-1/3

Table S2. **Contributions of all major elements to the total polarized nucleon number.**

Element	Mass fraction in crust	Mass fraction in mantle	Natural isotopes	Isotope abundance	Spin type	Total num. of atoms	Total num. of isotope	Net nucleon per atom	Polarized spin num.
Oxygen	46%	44%	^{17}O	0.038%	n	5.9×10^{49}	2.2×10^{46}	$+1$	$+3.5\times10^{35}$
Silicon	28%	21%	^{29}Si	4.7%	n	1.6×10^{49}	7.6×10^{47}	$+1$	$+7.3\times10^{36}$
Aluminum	8%	2.5%	^{27}AL	100%	p	1.9×10^{48}	1.9×10^{48}	$+1$	$+5.6\times10^{37}$
Iron	5%	6%	^{57}Fe	2.2%	n	3.3×10^{48}	7.4×10^{46}	$-1/3$	-3.9×10^{34}
Calcium	4%	2.5%	^{43}Ca	0.135%	n	1.4×10^{48}	1.9×10^{45}	1	$+1.8\times10^{34}$
Sodium	3%	<1%	^{23}Na	100%	p	2.6×10^{47}	2.6×10^{47}	$-3/5$	-3.4×10^{36}
Potassium	3%	<1%	^{39}K	93.3%	p	1.4×10^{46}	1.3×10^{46}	$-3/5$	-2.9×10^{34}
			^{41}K	6.7%	p	1.4×10^{46}	9.4×10^{44}	$-3/5$	-1.2×10^{33}
Magnesium	2%	23%	^{25}Mg	10%	n	2.0×10^{49}	2.0×10^{48}	$+1$	$+1.4\times10^{37}$

Table S3. **Geophysical abundance model uncertaintie.**

	Spin type	Atomic total number	Atomic number uncertainty	Polarized spin number	Polarized spin uncertainty
^{17}O	n	2.2×10^{46}	1.3×10^{45}	$+3.5\times10^{35}$	2.1×10^{34}
^{29}Si	n	7.6×10^{47}	4.6×10^{46}	$+7.3\times10^{36}$	4.5×10^{35}
^{27}Al	p	1.9×10^{48}	1.2×10^{47}	$+5.6\times10^{37}$	3.4×10^{36}
^{57}Fe	n	7.4×10^{46}	4.5×10^{45}	-3.9×10^{34}	2.4×10^{33}
^{43}Ca	n	1.9×10^{45}	1.2×10^{44}	$+1.8\times10^{34}$	1.1×10^{33}
^{23}Na	p	2.6×10^{47}	1.6×10^{46}	-3.4×10^{36}	2.1×10^{35}
^{39}K	p	1.3×10^{46}	7.9×10^{44}	-2.9×10^{34}	1.8×10^{33}
^{25}Mg	n	2.0×10^{48}	1.2×10^{47}	$+1.4\times10^{37}$	8.5×10^{35}
Proton	p	–	–	5.3×10^{37}	3.4×10^{36}
Neutron	n	–	–	2.2×10^{37}	9.6×10^{35}

Table S4. **Nuclear model uncertainties.**

Isotope	^{17}O	^{29}Si	^{27}Al	^{57}Fe	^{43}Ca	^{23}Na	^{39}K	^{25}Mg
Valence nucleon	n	n	p	n	n	p	p	n
Schmidt model	$+1/5$	$+1$	$+1/5$	$-1/3$	$1/7$	$-1/5$	$-1/5$	$1/5$
Semiempirical EV	$+0.198$	$+0.290$	$+0.100$	-0.047	$+0.098$	$+0.104$	-0.161	$+0.089$
Minimal FT	$+0.199$	$+0.711$	$+0.151$	-0.217	$+0.125$	-0.052	-0.181	$+0.155$
Preferred semi-empirical FT	$+0.199$	$+0.677$	$+0.145$	-0.203	$+0.123$	-0.034	-0.179	$+0.149$
Projection uncertainty	4×10^{-4}	0.145	0.021	0.059	0.009	0.062	0.008	0.022
Resulting polarized spin uncertainty	7.0×10^{32}	1.1×10^{36}	5.9×10^{36}	6.9×10^{33}	1.1×10^{33}	1.1×10^{36}	1.1×10^{33}	1.5×10^{36}
